# *Bacillus subtilis* ensures high spore quality in competition with *Salmonella* Typhimurium via the SigB-dependent pathway

**DOI:** 10.1093/ismejo/wraf052

**Published:** 2025-03-18

**Authors:** Eli Podnar, Kristina Dendinovic, Tjaša Danevčič, Bram Lories, Eva Kovačec, Hans Steenackers, Ines Mandic-Mulec

**Affiliations:** Department of Microbiology, Biotechnical Faculty, University of Ljubljana, Ljubljana 1000, Slovenia; Department of Microbiology, Biotechnical Faculty, University of Ljubljana, Ljubljana 1000, Slovenia; Department of Laboratory Medicine, Medical University of Vienna, Vienna, Austria; Department of Microbiology, Biotechnical Faculty, University of Ljubljana, Ljubljana 1000, Slovenia; Department of Microbial and Molecular Systems, Centre of Microbial and Plant Genetics (CMPG), KU Leuven, Leuven 3001, Belgium; Department of Microbiology, Biotechnical Faculty, University of Ljubljana, Ljubljana 1000, Slovenia; Agricultural Institute of Slovenia, Ljubljana, Slovenia; Department of Microbial and Molecular Systems, Centre of Microbial and Plant Genetics (CMPG), KU Leuven, Leuven 3001, Belgium; Department of Microbiology, Biotechnical Faculty, University of Ljubljana, Ljubljana 1000, Slovenia

**Keywords:** *Bacillus subtilis*, *Salmonella* Typhimurium, sporulation, probiotic, pathogen, SigB, T6SS, interspecies interactions

## Abstract

The interactions between beneficial bacteria and pathogens are understudied. Here we investigate the interactions between the probiotic strain *Bacillus subtilis* PS-216 and the pathogen *Salmonella* Typhimurium SL1344. We show here that the sporulation of *B. subtilis* is impaired when it competes with *S.* Typhimurium in a nutrient-depleted medium. The sporulation impairment in *B. subtilis* is mediated by the sigma factor B (SigB)-dependent general stress response, as the Δ*sigB* mutant remains blind to manipulative cues from *S.* Typhimurium. Furthermore, we show that decreased sporulation frequency in *B. subtilis* depends on cell–cell contact between the two species involving the *S.* Typhimurium Type VI Secretion System, whereas *B. subtilis* uses the SigB-dependent response to trade spore quantity for higher spore quality.

## Graphical Abstract

**Graphical Abstract:**
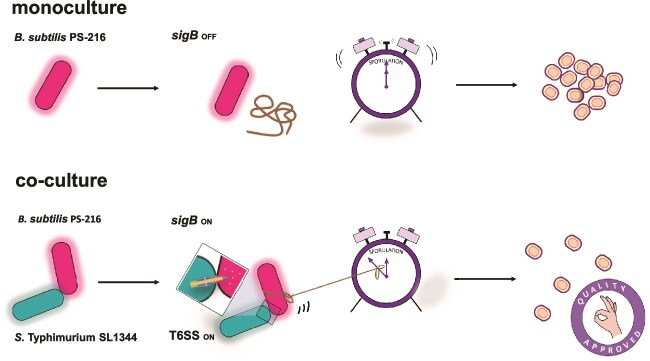

## Introduction

Microbial interactions are driving forces in microbial ecology [1] and play a crucial role in the development and management of biotechnological and biocontrol applications [2]. Although the number of studies addressing microbial social interactions is increasing, the role of interspecies interactions in shaping specific adaptive responses remains poorly understood.

Bacterial resistance to antibiotics emerged before their widespread use in medicine and agriculture. The increased use of antibiotics has driven the evolution of resistance mechanisms, which is especially problematic in pathogenic bacteria. As pathogens circulate between hosts and the environment, the environment has become a key reservoir of resistance genes, facilitating their acquisition by bacteria upon environmental exposure [3].

*Salmonella enterica* serovar Typhimurium (*S.* Typhimurium), a well-known pathogen, that causes diarrheal disease, is among the top most problematic carriers of antibiotic resistance [4]. Although *S.* Typhimurium is primarily recognized as a foodborne pathogen, it is also widespread in the environment, including soil [5], surface water [6], and plants [7]. Additionally, it can survive on abiotic surfaces causing issues in both industrial and domestic setting [8]. Survival outside a host requires rapid adaptation to various stress factors, such as low nutrient availability, enabling this pathogen to persist for a longer period and increasing its chances of re-entry into the food chain [5]. Studies have shown that *S.* Typhimurium can spread from soil to plants, including a variety of crops, posing the risk for consumers. Due to its wide host and habitat range *Salmonella* represents a major public health concern [9].

The use of probiotics offers an alternative strategy to combat the growing problem of antimicrobial resistant pathogens. *Bacillus subtilis* is a Gram-positive bacterium beneficial to plants and animals, known for its ability to produce various antimicrobial compounds [10]. It is mostly associated with soil and rhizosphere, but is also a member of the human and animal microbiota [11, 12]. *B. subtilis* is capable of inhibiting common pathogens such as *Staphylococcus aureus* [13], *Campylobacter jejuni* [14–16], and *Salmonella* Typhimurium [17]. In addition, the ability of *B. subtilis* to produce a variety of biologically active molecules, such as extracellular enzymes, vitamins, and antibiotics, expands the range of its applications [18], and makes it attractive for industrial use [19]. Moreover, as a spore forming bacterium, *B. subtilis* easily survives harsh environmental conditions through endospore formation. This resilience also contributes to its growing popularity as a probiotic for both humans and animals [20, 21].

Endospores are differentiated dormant cells that are highly resistant to a variety of environmental stressors. Spore development is induced when nutrients are lacking and growth is not favoured [22]. This highly regulated process is initiated via phosphorylation of the Spo0A transcription factor through a complex phosphorelay system [23, 24]. When Spo0A-P reaches a critical concentration, it binds to sporulation promoters and triggers the transcription of the associated sporulation genes [25]. After this stage, the cell is committed to spore development for several hours and is unable to grow until the sporulation process is completed. Therefore, *B. subtilis* has evolved numerous checkpoints and mechanisms to control premature commitment to sporulation [26]. For example, upon nutrient and energy stress, *B. subtilis* initially induces the general stress response (GSR) controlled by the sigma B transcription factor (SigB) (reviewed in [27]). SigB initiates a rapid response to environmental stressors [28–32] and temporarily inhibits sporulation by interfering with the Spo0A activity [33, 34]. However, starvation signals further evoke the Spo0A activity which in turn triggers sporulation in *B. subtilis* [35].

Recent work shows that interspecies competition can influence the sporulation of *B. subtilis*. For example, *Pseudomonas chlororaphis* PCL1606 stimulates sporulation in a contact-dependent manner by inducing specific histidine kinases, KinA and KinB, which are required for sporulation [36]. Siderophores pirated by *B. subtilis* from other species, such as *Escherichia coli*, also induce the sporulation process [37]. In general, studies have shown that *B. subtilis* upregulates sporulation in the presence of other bacteria, mainly for its protection against competitors [38–40].

Here we explore the influence of the common enteropathogen *Salmonella* Typhimurium [41] on the sporulation of *B. subtilis*. In contrast to prior studies utilizing other competitors, we show that *Salmonella* impairs the sporulation of *B. subtilis* and that in co-culture with *S.* Typhimurium, *B. subtilis* sporulation is affected through the SigB-dependent general stress response. Our results show that the impairment in sporulation is contingent on a sufficiently high *Salmonella* density and the presence of an active Type 6 Secretion System (T6SS) in *Salmonella*. These findings contribute to the understanding of the molecular links underlying social and adaptive strategies that shape the dynamics of microbial communities.

## Materials and methods

### Bacterial strains and strain construction

The strains used in this study are listed in Supplementary Table 1. Specific reporter fusions and gene deletions were inserted into the recipient strains by transformation. *B. subtilis* recipient strains were obtained using a standard transformation protocol in which the *B. subtilis* PS-216 [42] recipient strain was transformed with added DNA (plasmid or PCR product) [43]. Transformants were plated on LB agar plates with the appropriate antibiotics: erythromycin (Ery) 10 μg/ml, kanamycin (Kn) 50 μg/ml, or spectinomycin (Sp) 100 μg/ml.

*B. subtilis* PS-216 strains labelled with the red fluorescent protein mKate2, whose gene is linked to a constitutive P*_hyspank_* or P*_43_* promoter integrated into two different loci, *amyE*::P*_hyspank_-mKate2* [44–46] and *sacA*::P*_43_-mKate2,* were used.

To assay sporulation initiation, we generated the plasmid pEM1121 (Supplementary Table 2) carrying a P*_spo0A_*-*yfp* reporter fusion. Briefly, the P*_spo0A_* promoter region was PCR-amplified from *B. subtilis* PS-216 WT using the P*_spo0A_*-F (*EcoR*I) and P*_spo0A_*-R (*Hind*III) primer pair (Supplementary Table 3). The PCR product was then digested with *EcoR*I and *Hind*III restriction enzymes and ligated into the previously digested plasmid pKM3 [47] to obtain the plasmid pEM1121 (Supplementary Table 2). The plasmid pKM3 was digested with *EcoR*I and *Hind*III restriction enzymes to remove the P*_spoIIQ_* promoter region from the original vector. Plasmid pEM1121 was further transformed into *B. subtilis* PS-216 *sacA*::P*_43_*-*mKate2* (BM1629 [48]), producing the *B. subtilis* BM1996 strain.

The *B. subtilis* PS-216 Δ*sigB* mutant strain was generated by amplifying the erythromycin inactivated *sigB* gene from the genomic DNA of *B. subtilis* BKE24610 using the 5pL-sigB and 3pR-sigB primers [49] (listed in Supplementary Table 3). The PCR product of the erythromycin inactivated *sigB* gene was then transformed into the recipient strains *B. subtilis* PS-216 WT and *B. subtilis* PS-216 *amyE*::P*_hyspank_-mKate2* (BM1097 [44]) to obtain the *B. subtilis* BM1930 and BM1931 strains, respectively. The plasmid pMS17 [48] was transformed into *B. subtilis* PS-216 Δ*sigB* to generate *B. subtilis* PS-216 Δ*sigB sacA*::P*_43_-mKate2* (BM1992) reporter stain.

The plasmid pKM3 (Supplementary Table 2) carrying the P*_spoIIQ_-yfp* reporter fusion was transformed into *B. subtilis* PS-216 Δ*sigB sacA*::P*_43_-mKate2* (BM1956) strain to obtain the BM2017 strain.

*S.* Typhimurium SL1344 (WT) [50] and *S.* Typhimurium ATCC 14028 [51] were fluorescently labelled via the *gfpmut3* gene expressed from a plasmid [52, 53].

The *S.* Typhimurium SL1344 Δ*clpV,* Δ*hcp,* Δ*cpxA,* Δ*cpxP,* Δ*cpxR*, and Δ*rpoE* mutant strains were generated by phage P22 transduction. Donor strains were obtained from the *S.* Typhimurium ATCC 14028 background [54]. Briefly, 500 μl of donor strains were mixed with different amounts of phage (5 μl and 20 μl) and ~ 6 ml of TopAgar containing 8 g/L LB agar, 5 mM CaCl_2_, and 10 mM MgSO_4_, and poured on LB agar. Plates were checked for the presence of plaques the next day and the TopAgar was scraped off and pushed through a syringe and needle (22 G). The samples were centrifuged for 10 min at 13 000 g and the supernatants were transferred to a new tube to which a few drops of chloroform were added. After precipitation, the upper, clear supernatant was transferred to a new tube and mixed with 500 μl of the recipient strain, *S.* Typhimurium SL1344 WT. After centrifugation for 5 min at 5000 g, the pellet was dissolved in 100 μl of LB supplemented with 5 mM CaCl_2_, 10 mM MgSO_4_, and different amounts of phage (5 μl and 20 μl). The mixture was plated on LB supplemented with 20 mM EGTA and kanamycin for *S.* Typhimurium SL1344 Δ*clpV* and Δ*hcp* mutant strains or ampicillin for *S.* Typhimurium SL1344 Δ*cpxA,* Δ*cpxP,* Δ*cpxR,* and Δ*rpoE* mutant strains.

To determine the T6SS activity of *Salmonella*, we constructed a reporter fusion plasmid to measure the expression of *clpV.* Hereto, we inserted the promoter region of *clpV* upstream of the promoter-less *gfpmut3* gene in the pFPV25 backbone [52] using primers described in [55]. The construct was verified by PCR and sequence analysis.

### Growth conditions

To prepare overnight cultures, bacterial strains were grown in tryptic soy broth (TSB, Conda, Spain) supplemented with appropriate antibiotics at 37°C and shaken at 200 rpm for 16 h. The antibiotic concentrations in the medium were as follows: Cm 10 μg/ml, Kn 50 μg /ml, Ery 10 μg /ml, Sp 100 μg /ml, and Amp 100 μg /ml.

As described previously [17], overnight cultures were centrifuged for 10 min at 10 000 g, the supernatant discarded and the pellet re-suspended in 20-fold diluted (1/20) TSB medium. Suspensions of different *S.* Typhimurium strains were then diluted to OD_650_ ~ 0.1 absorbance units (a.u.), and suspensions of different *B. subtilis* strains were diluted to OD_650_ ~ 0.2 a.u. to obtain ~10^7^ cells/ml. *S.* Typhimurium was mixed with *B. subtilis* in a 1:1 ratio (V:V) and 100 μl of each co-culture sample was transferred to the wells of a 96-well F-bottom microtiter plate and incubated further for 24 h (or other time points if indicated) at 37°C under static conditions. In experiments in which different cell densities of *S.* Typhimurium were tested against *B. subtilis,* the initial inoculum containing 10^7^ cells/ml was diluted 10, 100, and 1000 times to obtain 10^6^, 10^5^, 10^4^ cells/ml, respectively. Monocultures were prepared in a 1:1 ratio with 1/20 TSB medium as control. The prepared monoculture and co-culture samples had an initial density before dilution ~10^7^ cells/ml for both bacteria, with the number of cells per species kept the same in the inoculum for monoculture and co-culture.

To estimate the cell numbers at the beginning and end of the experiment in monocultures and co-cultures, whole samples were disrupted by vigorous pipetting and vortexing. Disintegration of cell clumps into separate cells has been verified under the microscope.

### Estimation of the sporulation frequency in *B. *subtilis**

At different times of incubation, the total cell counts and the spore fraction of *B. subtilis* cells were determined by CFU/ml. To determine the number of spores, the samples were incubated for 30 min at 80°C and then plated on LB agar plates supplemented with the appropriate antibiotics. The sporulation frequency (SF) was then calculated by dividing the number of spores by the total cell count.

### Isolation of spores and estimation of the relative spore survival in *B. *subtilis**

*B. subtilis* WT and the Δ*sigB* mutant monoculture and co-culture samples were prepared as described above and incubated for 24 h at 37°C. To isolate the spores, the samples were incubated for 30 min at 80°C and then centrifuged three times for 10 min at 10 000 g. The supernatant was discarded and the pellet re-suspended in saline solution. Glycerol was then added in final concentration of 11% to store the spores at −20°C for further experiments.

The isolated spores were exposed to high temperature of 100°C for 30 min and then plated on LB plates. The relative spore survival was then calculated as the number of spores that survived the treatment divided by the total number of isolated spores before the treatment.

### Determination of the type of competition between *B. subtilis* and *S.* Typhimurium

#### Determination of the effect of conditioned medium on the sporulation

*S.* Typhimurium SL1344 monoculture and co-culture of *S.* Typhimurium SL1344 with *B. subtilis* PS-216 were prepared as described above and incubated for 24 h at 37°C. After incubation, the samples were centrifuged at 10 000 g for 10 min. To prepare conditioned medium, the supernatants were filtered through a filter with a pore diameter of 0.2 μm. Finally, the conditioned medium was mixed at a 1:1 (V/V) ratio with the *B. subtilis* strain prepared as described previously. The SF was calculated after 24 h of incubation.

#### Determination of the effect of lysed *S.* Typhimurium cells on the sporulation

Overnight culture of *S.* Typhimurium SL1344 was prepared in 1/20 TSB medium at 37°C and 200 rpm and then autoclaved at 121°C. The autoclaved *S.* Typhimurium culture was co-cultured with *B. subtilis* strain as described above. The SF was calculated after 24 h of incubation.

#### Transwell assay

The cultures were prepared as described above. An insert with a translucent PET membrane with a pore diameter of 0.4 μm was inserted into the tested wells (6-well plates, Greiner). First, 2.5 ml of *S.* Typhimurium culture was transferred into the well and then an insert containing 2.5 ml of *B. subtilis* culture was immersed into the well. The SF was calculated after 24 h of incubation.

### Visualization of *B. subtilis* sporulation

After 16 and 24 h of incubation, the samples were first disrupted by pipetting and 15 μl of each sample was transferred to glass slides (10 wells/ 6 mm). The slides were previously prepared by adding 10 μl of 0.05% poly-L-lysine to the well and air-dried at room temperature for 1.5 h as previously described [56]. After 15 min of samples incubation at room temperature, the excess liquid was aspirated. The samples were then rinsed with 15 μl of PBS buffer to remove unattached cells and dried for 10 min at room temperature. Finally, 2 μl of SlowFade Gold reagent was added to prevent fluorescence fading and the slides were covered with coverslips.

Visualization of *B. subtilis* PS-216 cells constitutively labelled with P*_43_-mKate2,* carrying P*_spoIIQ_*-*yfp* was performed. DIC microscopy allowed visualization of unlabelled *S.* Typhimurium cells in co-culture samples (not shown). Excitation of YFP was performed at 488 nm. The variable dichroics were set to reflect the emitted light in the range from 400–580 nm, which allowed to effectively capture fluorescence of YFP in the range of 500 to 580 nm by GaAsP PMT detector. Excitation of the red fluorescence protein (mKate2) was performed at 561 nm and the emitted fluorescence was captured in the range of 580 to 700 nm by GaAsP PMT detector. The laser intensities and GaAsP PMT detector gain were 4.5% and 650 V and 4% and 800 V for YFP and mKate2, respectively. The pinhole size was 55 μm. The wells on the slide were scanned with a 100 X / 0.4 N.A. objective and the captured images were 1024 × 1024 pixels with 16-bit colour depth.

Zen 2.3 Software (Carl Zeiss) was used for image processing and image noise was reduced by a single pixel filter (threshold = 1.5).

### Expression of different reporter strains

#### Bulk fluorescence measurements

The strains were prepared as described above in and then inspected as described previously [17]. Briefly, 100 μl of monocultures and co-cultures were allocated to at least three technical replicates in the wells of a 96-well black transparent bottom microtiter plate and incubated for 24 h at 37°C statically in a Cytation 3 imaging reader (BioTek, USA). To monitor gene expression, the fluorescence intensity of YFP was measured by excitation at 500 nm and emission at 530 nm, whereas the fluorescence intensity of mKate2 with excitation at 570 nm and emission at 620 nm. The gains of both fluorescence intensities were set to 100 and were measured every half hour. In unmarked samples, we measured both fluorescence intensities to obtain background values. Both values were then subtracted from the corresponding fluorescence intensities of the tagged samples. The mKate2 fluorescence intensity of the monoculture that represented the maximum value was set to a value of 1 and the remaining mKate2 fluorescence intensities were divided by the highest mKate2 fluorescence intensity. The YFP fluorescence intensity was then divided by the normalized mKate2 fluorescence intensity to obtain the fraction of activated cells.

#### Single cell measurements

To measure the activity of the P*_spoIIQ_* at the single-cell level, static monocultures and co-cultures in 1/20 TSB medium were prepared as described above. Three biological replicates were prepared for each promoter and each biological replicate consisted of two duplicates. The P*_spoIIQ_* activity was measured after 16 h and after 24 h. Prior to this, the samples were disrupted by vigorous pipetting and vortexing. Afterwards, 10 000 cells were measured with FACS Melody Cell Sorter (BD). Gene expression was analysed with FlowJo software. First, singlets were identified based on the forward and side scattering. Second, *B. subtilis* cells were identified based on the red fluorescence of constitutively expressed

P*_43_*-*mKate2*. Subsequently, the activity of the P*_spoIIQ_* was quantified by measuring the fluorescence intensity of the yellow fluorescent protein [17].

### Data presentation and statistical analysis

All experiments were performed in at least three independent biological replicates. The datasets obtained by CLSM are presented as one of the most representative images using Zen imaging software. Furthermore, statistical analysis and data presentation were performed in Origin program. Mean values and standard deviation (or standard error) were calculated from the obtained datasets and presented in the figures. Student’s t-test or one-way analyses of variance (ANOVA) with accompanying Tukey's post hoc test were used to compare means, and compared samples that showed a *P* value <0.05 were considered statistically different.

## Results

### *B. subtilis* sporulation is impaired in co-culture with *S.* Typhimurium

**Figure 1:**
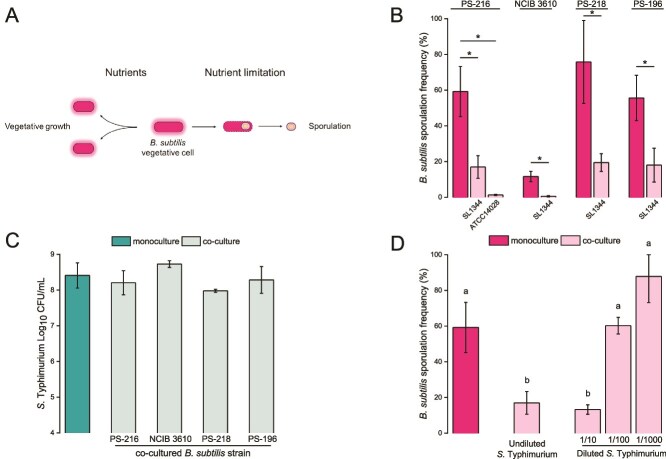
***S.* Typhimurium impairs *B. subtilis* sporulation in a density-dependent manner.** (A) *B. subtilis* shifts between two lifestyles: It grows when nutrients are available and during starvation it differentiates into dormant spores. (B) Sporulation frequency (SF) of different *B. subtilis* strains in monoculture and co-culture with *S.* Typhimurium SL1344 and ATCC 14028. (C) Cell counts of *S.* Typhimurium SL1344 in monoculture and co-culture with different *B. subtilis* strains. (D) SF of *B. subtilis* PS-216 in monoculture and in co-culture with undiluted (10^7^ cells/ml), 10x diluted (10^6^ cells/ml), 100x diluted (10^5^ cells/ml) and 1000x diluted (10^4^ cells/ml) initial inocula of *S.* Typhimurium SL1344. The SF for *B. subtilis* and cell counts for *S.* Typhimurium were determined after 24 h of static incubation at 37°C in 1/20 TSB medium. Data for (B), (C), and (D) are presented as mean values and error bars represent standard deviation of the mean values of three biologically independent experiments. Student’s t-test was performed to compare the means of SF and cell counts between monoculture and co-culture for (B) and (C) where statistically significant differences were determined (^*^P < 0.05). One-way ANOVA with Tukey's post hoc test (*P* < 0.05) was performed to compare the means of SF between monoculture and different co-cultures for (D), where different letters above the columns indicate a significant difference between samples.

Nutrient-depleted conditions lead to the formation of metabolically inactive *B. subtilis* spores [57] (Fig. 1A). This adaptive process has also been reported to serve as a defence mechanism during competition with other bacteria [36–40]. Here we focus on the sporulation frequency (SF) of *B. subtilis* in a previously characterized interaction with *S.* Typhimurium SL1344 [17]. We performed the experiments in nutrient-depleted medium (1/20 TSB), which promotes *B. subtilis* sporulation. Our results showed that *B. subtilis* PS-216 co-cultured with *S.* Typhimurium SL1344 exhibited a lower SF compared to monoculture conditions. This phenomenon was also observed in other *B. subtilis* strains, namely the laboratory wild-type strain *B. subtilis* NCIB 3610 and two soil isolates, *B. subtilis* PS-218 and *B. subtilis* PS-196 (Fig. 1B). Moreover, in addition to *S.* Typhimurium SL1344, *S.* Typhimurium ATCC 14028 also decreased SF of *B. subtilis* PS-216 (Fig. 1B). Therefore, impaired sporulation is a widespread and not a strain specific phenomenon, occurring across different *B. subtilis* and *S.* Typhimurium strains. Cell number may also play an important role in interspecies interactions as some effects may be cell density dependent. *S.* Typhimurium reaches similar densities in co-cultures with different *B. subtilis* strains in 1/20 medium (Fig. 1C). However, SF was not decreased if the *S.* Typhimurium inoculum was diluted 100- or 1000-fold (Fig. 1D), suggesting that impaired sporulation by *S.* Typhimurium is cell density dependent and does not occur at low pathogen densities.

**Figure 2:**
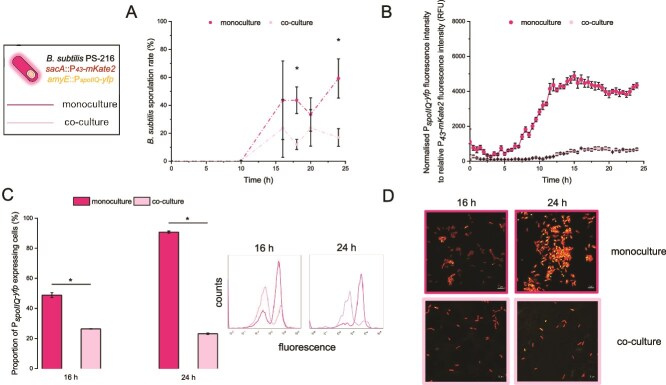
**Influence of *S.* Typhimurium on *B. subtilis* PS-216 sporulation dynamics.** (A) *B. subtilis* SF was determined after 10, 16, 18, 20 and 24 h of static growth at 37°C in 1/20 TSB medium for monoculture and co-culture. (B) Bulk measurements of the P*_spoIIQ_*-*yfp* promoter activity in *B. subtilis* PS-216 monoculture and co-culture with *S.* Typhimurium SL1344. Only one out of three biologically independent experiments is shown on the panel (performed in six technical replicates). Measurements were performed every half hour for 24 h in 1/20 TSB medium. Data are presented as mean values and error bars represent standard error of the mean values. (C) Flow measurements and (D) microscopic images of single-cell transcriptional activity of the P*_spoIIQ_*-*yfp* of *B. subtilis* PS-216 in monoculture and co-culture with *S.* Typhimurium SL1344 were performed after 16 h and 24 h of static growth at 37°C in 1/20 TSB medium. Data are presented as mean values and error bars represent standard deviation of the mean values of three biologically independent experiments. Student’s t-test was performed to compare the means of SF and the proportion of P*_spoIIQ_*-*yfp* expressing cells between monoculture and co-culture, where statistically significant differences were determined (^*^P < 0.05). One representative repeat of at least three biologically independent experiments (performed in at least two technical replicates) is shown for both (C) and (D). In the microscopic images, the scale bar represents 5 μm.

We also determined the sporulation dynamics (Fig. 2A, Supplementary Fig. 1). Sporulation of PS-216 started after 10 h of incubation in both monoculture and co-culture, but consistently reached higher SFs in monoculture conditions, reaching up to 60% at the end of the experiment. In contrast, in the presence of *Salmonella*, sporulation stagnated around 20%. Total cell counts and spore counts are shown in Supplementary Fig. 1. To visualize and measure sporulation dynamics, the *B. subtilis* PS-216 P*_spoIIQ_*-*yfp* P*_43_-mKate2* reporter strain [48] was grown with *S.* Typhimurium or alone, using the expression of the *spoIIQ* gene (expressed in the forespore [58]) as a sporulation marker. This fluorescent reporter strain showed similar monoculture and co-culture sporulation dynamics at the population level as our previous assay using traditional plating methods, confirming the utility of this reporter strain for determining sporulation dynamics (Fig. 2B). Subsequently, we measured and visualized the P*_spoIIQ_*-*yfp* activity at the single cell level after 16 h and 24 h of incubation using flow cytometry and confocal laser scanning microscopy (Fig. 2C, Fig. 2D). These assays confirm that sporulation genes were induced in smaller subpopulation in co-culture compared to monoculture.

To test whether *S.* Typhimurium negatively affects sporulation before stage II, we constructed a reporter strain carrying the promoter of the master regulator *spo0A* fused to YFP. The P*_spo0A_-yfp* transcriptional activity was initially higher in the monoculture than in the co-culture, confirming that the sporulation impairment occurs early during spore development. As expected, at later stages, from 17 h onwards, when P*_spo0A_-yfp* activity in monoculture dropped rapidly, the P*_spo0A_-yfp* activity in the co-culture also dropped, but not as intensely as in monoculture (Supplementary Fig. 2).

### *S.* Typhimurium affects sporulation through the transcriptional factor SigB leading to higher spore quality

**Figure 3:**
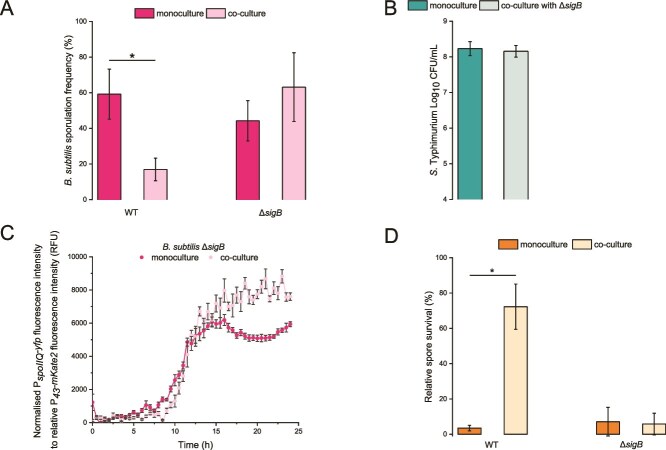
**Impairment of *B. subtilis* PS-216 sporulation is dependent on the SigB transcription factor.** (A) SF of the *B. subtilis* PS-216 Δ*sigB* mutant in monoculture and co-culture with *S.* Typhimurium SL1344. SF was determined after 24 h of static incubation at 37°C in 1/20 TSB medium (B) cell counts of *S.* Typhimurium SL1344 in monoculture and co-culture with *B. subtilis* PS-216 Δ*sigB* mutant after 24 h of static incubation at 37°C in 1/20 TSB medium. Data for (A) and (B) are presented as mean values and error bars represent standard deviation of the mean values of three biologically independent experiments. Student’s t-test was performed to compare the means of SF and cell counts between monoculture and co-culture, where statistically significant differences were determined (^*^P < 0.05). (C) Bulk measurements of the P*_spoIIQ_*-*yfp* promoter transcriptional activity in *B. subtilis* PS-216 Δ*sigB* mutant monoculture and co-culture with *S.* Typhimurium SL1344. Only one out of three biologically independent experiments is shown on the panel (performed in six technical replicates). Measurements were performed every half hour for 24 h in 1/20 TSB medium. Data are presented as mean values and error bars represent standard error of the mean values. (D) *B. subtilis* PS-216 WT and the Δ*sigB* mutant relative spore survival in monoculture and co-culture with *S.* Typhimurium SL1344. Spore survival was determined after 30 min of incubation at 100°C and was divided by total spores. Data are presented as mean values and error bars represent standard deviation of the mean values of four biologically independent experiments. Student’s t-test was performed to compare the means between monoculture and co-culture, where statistically significant differences were determined (^*^P < 0.05).

In *B. subtilis*, the alternative SigB is responsible for sporulation inhibition under various stress conditions, including nutrient-limitation and energy stress. SigB acts at an early stage of spore development by interfering with Spo0A-P-dependent effects [33, 34]. SigB controls the GSR, which is a rapid and reversible response to different stressors including low nutrients [30, 59, 60]. In contrast, sporulation is an irreversible response following the induction of sporulation stage II genes [61]. Therefore, we tested whether competition with *Salmonella* affects sporulation via a SigB-dependent pathway. We compared the SF of the *B. subtilis* PS-216 WT and Δ*sigB* mutant in monoculture and co-culture. *S.* Typhimurium did not impair sporulation in the PS-216 Δ*sigB* mutant (Fig. 3A), indicating that pathogen-mediated disruption of sporulation is SigB-dependent. Furthermore, the Δ*sigB* mutant did not reduce the growth of the pathogen in co-culture, confirming that the lack of negative impact on sporulation is not due to a lower cell counts of the pathogen (Fig. 3B). The spore count and the total number of Δ*sigB* cells of *B. subtilis* is shown in Supplementary Fig. 3.

To test whether SigB affects the expression of sporulation genes, we introduced the P*_spoIIQ_*-*yfp* reporter into the PS-216 Δ*sigB* background and tested promoter activity in monoculture and co-culture with *S.* Typhimurium. Again, we did not observe any repression of the P*_spoIIQ_* promoter activity in the mutant. In contrast, the promoter activity in the P*_spoIIQ_-yfp* reporter in the co-cultured mutant was initially comparable to that in monoculture, and then even started to increase during the last stages of incubation (Fig. 3C). These results support the conclusion that *S.* Typhimurium impairs sporulation via a SigB-dependent pathway.

Recent reports show that *B. subtilis* sporulation can follow a quality-quantity trade-off [62, 63]. We test here whether the competition between *B. subtilis* and *S.* Typhimurium can also affect spore quality. We defined quality as the durability of spores at high temperatures (100°C). Therefore, we isolated spores after 24 h from monoculture and co-culture samples of *B. subtilis* WT (sporulation impairment in co-culture) and the Δ*sigB* mutant (no sporulation impairment). We then exposed the spores to 100°C for 30 min. The results showed that the WT spores produced in co-culture had a higher survival frequency at high temperatures than the spores from the monoculture. In addition, the increase in spore quality was dependent on SigB, as the spores of the co-cultured Δ*sigB* mutant do not show an increased tolerance to heat (Fig. 3D).

### Interactions between *B. subtilis* and *S.* Typhimurium are contact-dependent

**Figure 4:**
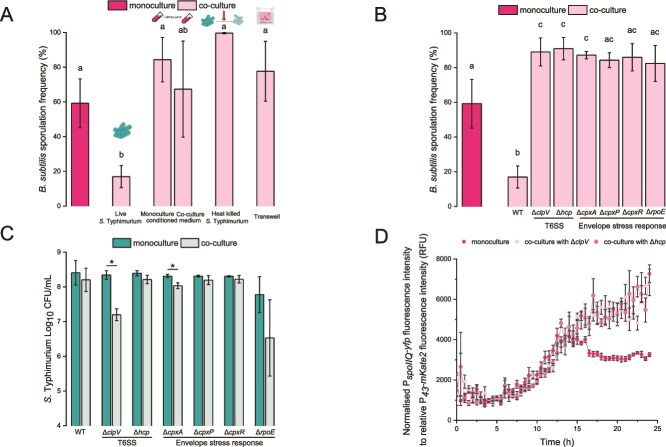
**Direct cell-to-cell contact and *S.* Typhimurium T6SS is needed for sporulation impairment.** (A) SF of *B. subtilis* PS-216 in monoculture and co-cultures with live and heat-killed *S.* Typhimurium SL1344, conditioned medium and in transwell assay. SF was determined after 24 h of static incubation at 37°C in 1/20 TSB medium. (B) SF of the *B. subtilis* PS-216 in monoculture and co-culture with *S.* Typhimurium SL1344 WT strain and mutants in T6SS and envelope stress response and (C) cell counts of *S.* Typhimurium SL1344 WT strain and mutants in T6SS and envelope stress response. SF and cell counts were determined after 24 h of static incubation at 37°C in 1/20 TSB medium. Data for (A), (B), and (C) are presented as mean values and error bars represent standard deviation of the mean values of three biologically independent experiments. One-way ANOVA with Tukey's post hoc test (*P* < 0.05) was performed to compare the means of SF between monocultures and co-cultures for (A) and (B), where different letters above the columns indicate a significant difference between samples. Student’s t-test was performed to compare the means of SF and cell counts between monoculture and co-culture for (C), where statistically significant differences were determined (^*^P < 0.05). (D) Bulk measurements of the P*_spoIIQ_*-*yfp* promoter transcriptional activity in *B. *subtilis** PS-216 monoculture and co-culture with the *S.* Typhimurium SL1344 Δ*clpV* and Δ*hcp* mutants. Measurements were performed every half hour for 24 h in 1/20 TSB medium. Only one out of three biologically independent experiments is shown on the panel (performed in six technical replicates). Measurements were performed every half hour for 24 h in 1/20 TSB medium. Data are presented as mean values and error bars represent standard error of the mean values.

We aimed to understand how *S.* Typhimurium triggers the SigB-dependent effect on sporulation frequency. We hypothesized that *S.* Typhimurium might act through either a) secretion of diffusible factors, b) shedding of its own cell components that act as a signal, or c) contact-mediated mechanisms*.* To test these predictions, we cultured *B. subtilis* PS-216 with conditioned medium from either *S.* Typhimurium SL1344 monoculture or co-culture, as the presence of *B. subtilis* may alter the compounds secreted by *Salmonella*. We also tested autoclaved (heat killed) *S.* Typhimurium SL1344 cells to evaluate the impact of the cell wall components on sporulation. Finally, we tested the effects of cell–cell contact by co-culturing both species in a transwell setup, where both bacteria were separated by a semi-permeable membrane. The results show that only live *Salmonella* decreased SF of *B. subtilis*, whereas conditioned medium or heat killed *S.* Typhimurium did not lower SF. Moreover, preventing cell-to-cell contact between the pathogen and *B. subtilis* also abolished the decrease in SF (Fig. 4A). Therefore, we conclude that direct cell-to-cell contact between viable bacteria is required for the sporulation impairment phenotype. The total cell counts and spore counts for the different co-cultures are shown in Supplementary Fig. 4.

We speculated that the contact-dependent Type 6 Secretion System (T6SS) might contribute to sporulation impairment. The T6SS, a complex contractile needle-like system, is used by Gram negative bacteria for the injection of toxins into competitors [64, 65]. To test this hypothesis, we co-cultured *B. subtilis* PS-216 with T6SS-defective *Salmonella* mutants and measured the SF of PS-216 after 24 h of incubation. These mutants with a deletion in *clpV* or *hcp*, respectively encoding the ATPase and the inner tube of the T6SS [64], did not decrease SF (Fig. 4B).

It has been shown that T6SS expression is mediated by the envelope stress response in *E. coli* and *Citrobacter rodentium* [66, 67]. *S.* Typhimurium has various systems that respond to changes to the membrane [68], with the best studied being the σ^E^ and the two component Cpx system. σ^E^ responds to stress at the outer membrane, whereas Cpx recognizes stress at the inner membrane [69]. In the Cpx system, CpxA is the sensor histidine kinase, whereas CpxR is the response regulator [70]. The third component of the Cpx system, CpxP, inhibits the activity of CpxA [71]. *S.* Typhimurium mutants defective in the envelope stress response (i.e. Δ*cpxA*, Δ*cpxP,* Δ*cpxR,* Δ*rpoE*) were also unable to reduce *B. subtilis* SF (Fig. 4B). The deletion in genes for T6SS and envelope stress response in *S.* Typhimurium result in a similar number of *B. subtilis* cells in co-culture (Supplementary Fig. 5).

Because the results can also be explained by reduced growth of the *Salmonella* mutants in co-cultures, we determined their cell density in co-cultures. Mutants Δ*clpV* and Δ*cpxA* were found to have reduced growth in co-cultures (Fig. 4C). However, other mutants, including T6SS-related mutants, showed comparable growth in co-culture to wild-type *S.* Typhimurium (Fig. 4C). This suggests that a complex, contact-dependent interplay between *B. subtilis* and *S.* Typhimurium, involving the pathogen’s T6SS and some envelope stress response genes, may be responsible in the sporulation impairment phenotype.

We then monitored the P*_spoIIQ_-yfp* expression of *B. subtilis* in co-culture with *S.* Typhimurium Δ*clpV* and Δ*hcp* mutants. The results showed that the P*_spoIIQ_-yfp* expression was comparable to that in the monoculture during the first 16 h. However, thereafter we detected an increase in the P*_spoIIQ_-yfp* expression in the co-culture with the two *S.* Typhimurium mutants (Fig. 4D). These results further confirm our findings that the T6SS-defective *S.* Typhimurium mutants do not impair *B. subtilis* sporulation.

Because *B. subtilis* requires iron for sporulation [72], we hypothesized that iron limitation may contribute to the T6SS-mediated sporulation impairment. To test this, we added FeCl₃ (0.05 and 0.1 mM) to 1/20 TSB medium and measured sporulation frequency. Under iron-rich conditions, sporulation of *B. subtilis* in co-culture with *S.* Typhimurium was no longer impaired (Supplementary Fig. 6).

## Discussion

*S.* Typhimurium is a good biofilm former, which allows it to survive in different environments [8] including non-host environments [5]. Its widespread environmental persistence poses a major challenge to pathogen control and contributes to the spread of antimicrobial resistance [41]. The persistence of *Salmonella* in soil has been found to largely depend on soil microbiota diversity, where greater diversity inhibits *Salmonella* invasion and abundance, whereas lower diversity promotes it [73]. Understanding the ecological perspective of *S.* Typhimurium and its interactions with other soil microorganisms, including *B. subtilis,* may contribute to understanding *S.* Typhimurium lifestyle outside the host and thus reduce the associated public health risks.

The endospore development of *B. subtilis* has been intensively studied for decades as spores are among the most resilient life forms on the planet. Endospores confer *B. subtilis* with increased tolerance to a range of stressors, including low and high temperatures, UV and γ-radiation, desiccation, harmful chemicals, predation, and nutrient depletion [22, 74, 75]. Therefore, sporulation is mainly induced in stress environments, including nutrient-depleted conditions. However, once *B. subtilis* enters stage II of sporulation (asymmetric septum formation), it cannot resume vegetative growth before sporulation is completed [61]. Consequently, *B. subtilis* has evolved several mechanisms to prevent premature entry into sporulation, which include activation of *spo0A* transcription and Spo0A phosphorylation [76, 77]. In addition, rapid entry into sporulation upon nutrient limitation is prevented via SigB. This stress response system senses nutrient and energy stress and postpones sporulation by acting as a transcriptional inhibitor of spore development [34].

**Figure 5:**
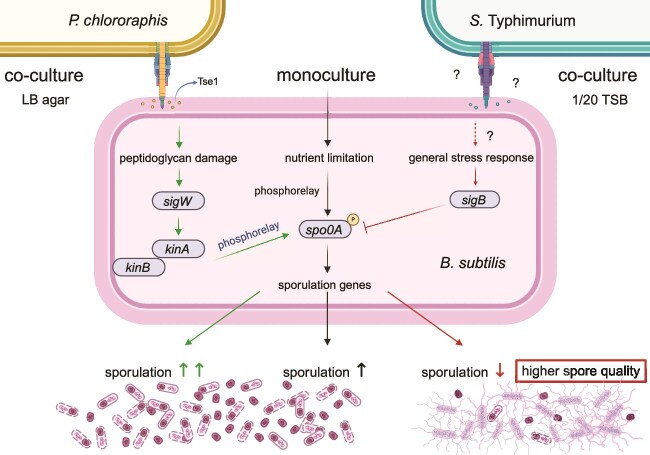
**Model of the *B. subtilis* survival strategies of in competition with *P. chlororaphis* and *S.* Typhimurium.** During interaction with *P. chlororaphis* on LB agar*, B. subtilis* responds to Tse1-T6SS-mediated envelope disturbance by upregulating sporulation via SigW, KinA, and KinB. Under nutrient-depleted conditions in 1/20 TSB, *B. subtilis* sporulates in response to starvation. However, co-cultivation with *S.* Typhimurium impairs sporulation. This effect is mediated by the T6SS of *S.* Typhimurium, which in an unknown manner activates the SigB-dependent general stress response, resulting in formation of fewer but higher quality spores. Created in BioRender. Podnar, E. (2025) https://BioRender.com/x41m214.

Despite the importance of sporulation, knowledge of how interspecies social interactions influence spore development is still limited. Most published work highlights the induction of sporulation as a defence mechanism of *B. subtilis* against competitors such as *Myxococcus xanthus*, *E. coli*, and *P. chlororaphis* [36–40]. In contrast, our work shows that competition with the enteropathogen *S.* Typhimurium leads to an impairment in sporulation. The sporulation impairment requires pathogen’s T6SS system and close cell–cell interactions. Although the experiments are performed in liquid medium, under static conditions, both species form submerged biofilm enable mixing and thus facing the close contact between competitors [17]. This is consistent with our observation that T6SS of *S.* Typhimurium is involved in sporulation impairment and that the phenotype depends on cell–cell contact. T6SSs are found in 25% of Gram-negative species capable of releasing diverse effectors into bacterial and eukaryotic cells [78]. However, the knowledge of the T6SS attack mechanisms against *Bacillus* is limited [40, 79]. Recent study shows that the plant beneficial bacterium *P. chlororaphis* delivers the Tse1 toxin via T6SS to *B. subtilis* cells. Tse1 is a hydrolase that degrades *Bacillus* peptidoglycan and indirectly damages *Bacillus* membrane functionality and activates the SigW dependent pathway, which promotes sporulation. It was proposed that this response permits *B. subtilis* to defend against the toxicity of T6SS-mobilized Tse1 effector [40]. In contrast, the T6SS of *S.* Typhimurium induces the SigB-dependent general stress response in *B. subtilis*, impairing the commitment to sporulation with concomitant increase in spore quality. *S.* Typhimurium SL1344 strain we used here has only one known effector, Tae4, which is an amidase that cleaves the γ-d-glutamyl-l-meso-diaminopimelic acid amide bond of peptidoglycan [64]. Because the deletion of the *clpV* and *hcp* genes, which are essential for a functional T6SS, did not alter the fitness of either *S.* Typhimurium or *B. subtilis* in co-culture, we cannot claim at this point that T6SS mediates the attack of *B. subtilis* via toxin release. In Fig. 5, we summarized the current knowledge on interspecies interactions between *P. chlororaphis* or *S.* Typhimurium with *B. subtilis* and their impact on *B. subtilis* sporulation.

From an evolutionary perspective, sporulation is a conserved process in bacteria [80]. However, according to our results, prompt sporulation does not seem to be beneficial in the competition with *S.* Typhimurium. The impairment in sporulation gives *B. subtilis* an ecological advantage as the higher quality of the spores is ensured by affecting the SigB-dependent pathway. Although the deletion of the *sigB* gene does not negatively affect the growth of either species, it seems that low quantity and high-quality trade-off in spore development is an adaptive strategy of *B. subtilis* and less likely a manipulative strategy of *S.* Typhimurium to gain a competitive advantage. Although, we cannot completely rule out that *S.* Typhimurium rips benefits form inhibiting sporulation during prolonged co-incubations spanning beyond 24 h. The trade-off between spore quality and quantity has recently been hypothesized to promote the survival of *B. subtilis* under harsh conditions [62, 63]. As a result, the trade-off may result in *B. subtilis* having a better chance of reproducing when conditions improve. The quality-quantity trade-off is also consistent with our observation that SigB plays an important role in competition between the two species. For example, the Δ*sigB* mutants of *Bacillus cereus* have been shown to produce low-quality spores [81]. Thus, by impairing sporulation, SigB-dependent GSR also mediates a trade-off between quality and quantity during competition with *S.* Typhimurium. Also, *B. subtilis* interactions with fungus *Fusarium verticillioides* induce SigB-dependent stress response, resulting in the surfactin mediated antagonism against the fungus [82]. This result suggests that SigB-dependent regulatory response to competition may be more-wide spread, but more research is needed to clarify whether it is linked to quality-quantity trade-off, we observed in interactions with *S.* Typhimurium.

External conditions, such as medium composition, significantly influence spore properties. Divalent cations (Mn^2+^, Ca^2+^, Zn^2+^, Mg^2+^) enhance spore stability and yield [83]. It is likely that interspecies competition changes the availability of essential nutrients, including ions. This could be sensed by *B. subtilis* SigB activating pathway and consequently affects the spore quantity-quality trade off. In line with these findings, T6SS also plays a role in ion sequestration in some bacterial species [84]. Although the T6SS role in ion sequestration by *S.* Typhimurium needs to be tested in the future, it is intriguing that addition of iron to the nutrient limited medium bypasses the sporulation impairment triggered by *S.* Typhimurium. This suggests that competition for iron between *S.* Typhimurium and *B. subtilis* could alter the competition dynamics and consequently affect adaptive responses of *B. subtilis* (for discussion see Supplementary Fig. 6 and associated text).

At this point exact environmental factors and molecular cues triggering the *S.* Typhimurium T6SS dependent activation of the SigB general stress response in *B. subtilis* remain unclear. It will be important to test the role of specific T6SS effectors and/or nutrient signals to better define the molecular cues exchanged during interspecies competition.

Overall, our results reveal a response of *B. subtilis* to interspecific competition and nutrient limitation, in which it favours growth over sporulation at an earlier stage. Although sporulation has been considered as the primary defence mechanism of *B. subtilis*, here we show that co-culture with *S.* Typhimurium stimulates *B. subtilis* to adopt a strategy to impair sporulation. This work reveals new insights into the intricate dynamics of bacterial interactions dictated by SigB and the GSR of *B. subtilis*, leading to a change in the commitment of *B. subtilis* to sporulation and the production of higher quality spores during interspecies competition. This could be of great importance for understanding the ecology of this bacterium within microbial communities under nutrient-limited conditions.

## Supplementary Material

Supplementary_material_Podnar_et_al_2025_13_3_wraf052

## Data Availability

All the data are provided in the published article and its supplementary information files. Raw data of this study have been deposited in Figshare: https://doi.org/10.6084/m9.figshare.26526805

## References

[1] Ghoul M, Mitri S. The ecology and evolution of microbial competition. *Trends Microbiol* 2016;24:833–45. 10.1016/j.tim.2016.06.011 27546832

[2] Tshikantwa TS, Ullah MW, He F. et al. Current trends and potential applications of microbial interactions for human welfare. *Front Microbiol* 2018;9:1156. 10.3389/fmicb.2018.01156 29910788 PMC5992746

[3] Larsson DGJ, Flach CF. Antibiotic resistance in the environment. *Nat Rev Microbiol* 2022;20:257–69. 10.1038/s41579-021-00649-x 34737424 PMC8567979

[4] Rello J, Kalwaje Eshwara V, Lagunes L. et al. A global priority list of the TOp TEn resistant Microorganisms (TOTEM) study at intensive care: a prioritization exercise based on multi-criteria decision analysis. *Eur J Clin Microbiol Infect Dis* 2019;38:319–23. 10.1007/s10096-018-3428-y 30426331

[5] Winfield MD, Groisman EA. Role of nonhost environments in the lifestyles of *Salmonella* and *Escherichia coli*. *Appl Environ Microbiol* 2003;69:3687–94. 10.1128/AEM.69.7.3687-3694.2003 12839733 PMC165204

[6] Liu H, Whitehouse CA, Li B. Presence and persistence of *salmonella* in water: the impact on microbial quality of water and food safety. *Front Public Heal* 2018;6:159. 10.3389/fpubh.2018.00159 PMC5989457 29900166

[7] Agnès W, Isabelle VP, Anne-Marie CM. et al. Interactions of *Salmonella* with animals and plants. *Front Microbiol* 2014;5:791. 25653644 10.3389/fmicb.2014.00791 PMC4301013

[8] Steenackers H, Hermans K, Vanderleyden J. et al. *Salmonella* biofilms: an overview on occurrence, structure, regulation and eradication. *Food Res Int* 2012;45:502–31. 10.1016/j.foodres.2011.01.038

[9] Waldner LL, MacKenzie KD, Köster W. et al. From exit to entry: long-term survival and transmission of *Salmonella*. *Pathogens* 2012;1:128–55. 10.3390/pathogens1020128 25436767 PMC4235688

[10] Caulier S, Nannan C, Gillis A. et al. Overview of the antimicrobial compounds produced by members of the *Bacillus subtilis* group. *Front Microbiol* 2019;10:302. 10.3389/fmicb.2019.00302 30873135 PMC6401651

[11] Hong HA, Khaneja R, Tam NMK. et al. *Bacillus subtilis* isolated from the human gastrointestinal tract. *Res Microbiol* 2009;160:134–43. 10.1016/j.resmic.2008.11.002 19068230

[12] Barbosa TM, Serra CR, La Ragione RM. et al. Screening for *Bacillus* isolates in the broiler gastrointestinal tract. *Appl Environ Microbiol* 2005;71:968–78. 10.1128/AEM.71.2.968-978.2005 15691955 PMC546680

[13] Kimelman H, Shemesh M. Probiotic Bifunctionality of *Bacillus subtilis*—rescuing lactic acid bacteria from desiccation and antagonizing pathogenic *Staphylococcus aureus*. *Microorganisms* 2019;7:407. 10.3390/microorganisms7100407 31569575 PMC6843919

[14] Erega A, Stefanic P, Dogsa I. et al. Bacillaene mediates the inhibitory effect of *Bacillus subtilis* on *Campylobacter jejuni* biofilms. *Appl Environ Microbiol* 2021;87:e0295520. 10.1128/AEM.02955-20 33837012 PMC8174767

[15] Erega A, Stefanic P, Danevčič T. et al. Impact of *Bacillus subtilis* antibiotic bacilysin and *Campylobacter jejuni* efflux pumps on pathogen survival in mixed biofilms. *Microbiol Spectr* 2022;10:e01256–22. 10.1128/spectrum.02156-22 PMC9430781 35938811

[16] Šimunović K, Stefanic P, Klančnik A. et al. *Bacillus subtilis* PS-216 antagonistic activities against *Campylobacter jejuni* NCTC 11168 are modulated by temperature, oxygen, and growth medium. *Microorganisms* 2022;10:289. 10.3390/microorganisms10020289 35208741 PMC8875091

[17] Podnar E, Erega A, Danevčič T. et al. Nutrient availability and biofilm polysaccharide shape the bacillaene-dependent antagonism of *Bacillus subtilis* against *Salmonella* Typhimurium. *Microbiol Spectr* 2022;10:e01836–22. 10.1128/spectrum.01836-22 36342318 PMC9769773

[18] Zhang X, Al-Dossary A, Hussain M. et al. Applications of *Bacillus subtilis* spores in biotechnology and advanced materials. *Appl Environ Microbiol* 2020;86:e01096–20. 10.1128/AEM.01096-20 32631858 PMC7440806

[19] Su Y, Liu C, Fang H. et al. *Bacillus subtilis*: a universal cell factory for industry, agriculture, biomaterials and medicine. *Microb Cell Factories* 2020;19:1–12. 10.1186/s12934-020-01436-8 PMC7650271 32883293

[20] Cutting SM . *Bacillus* probiotics. *Food Microbiol* 2011;28:214–20. 10.1016/j.fm.2010.03.007 21315976

[21] Ritter AC, Paula A, Correa F. et al. Characterization of *Bacillus subtilis* available as probiotic characterization of *Bacillus subtilis* available as probiotics. *J Microbiol Res* 2018;8:23–32.

[22] Setlow P . I will survive: DNA protection in bacterial spores. *Trends Microbiol* 2007;15:172–80. 10.1016/j.tim.2007.02.004 17336071

[23] Fujita M, Gonza E, Losick R. High- and low-threshold genes in the Spo0A regulon of *Bacillus subtilis*. *J Bacteriol* 2005;187:1357–68. 10.1128/JB.187.4.1357-1368.2005 15687200 PMC545642

[24] Tan IS, Ramamurthi KS. Spore formation in *Bacillus subtilis*. *Environ Microbiol Rep* 2014;6:212–25. 10.1111/1758-2229.12130 24983526 PMC4078662

[25] Hoch JA . Regulation of the phosphorelay and the initiation of sporulation in *Bacillus subtilis*. *Ann Rev Microbiol* 1993;47:441–65. 10.1146/annurev.mi.47.100193.002301 8257105

[26] Phillips ZEV, Strauch MA. *Bacillus subtilis* sporulation and stationary phase gene expression. *Cell Mol Life Sci* 2002;59:392–402. 10.1007/s00018-002-8431-9 11964117 PMC11337539

[27] Rodriguez Ayala F, Bartolini M, Grau R. The stress-responsive alternative sigma factor SigB of *Bacillus subtilis* and its relatives: an old friend with new functions. *Front Microbiol* 2020;11:1761. 10.3389/fmicb.2020.01761 33042030 PMC7522486

[28] Benson AK, Haldenwang WG. The σ(B)-dependent promoter of the *Bacillus subtilis sigB* operon is induced by heat shock. *J Bacteriol* 1993;175:1929–35. 10.1128/jb.175.7.1929-1935.1993 8458834 PMC204264

[29] Brigulla M, Hoffmann T, Krisp A. et al. Chill induction of the SigB-dependent general stress response in *Bacillus subtilis* and its contribution to low-temperature adaptation. *J Bacteriol* 2003;185:4305–14. 10.1128/JB.185.15.4305-4314.2003 12867438 PMC165770

[30] Zhang S, Haldenwang WG. Contributions of ATP, GTP, and redox state to nutritional stress activation of the *Bacillus subtilis* σB transcription factor. *J Bacteriol* 2005;187:7554–60. 10.1128/JB.187.22.7554-7560.2005 16267279 PMC1280325

[31] Hecker M, Pané-Farré J, Völker U. SigB-dependent general stress response in *Bacillus subtilis* and related gram-positive bacteria. *Ann Rev Microbiol* 2007;61:215–36. 10.1146/annurev.micro.61.080706.093445 18035607

[32] Bonilla CY . Generally stressed out bacteria: environmental stress response mechanisms in gram-positive bacteria. *Integr Comp Biol* 2020;60:126–33. 10.1093/icb/icaa002 32044998

[33] Reder A, Albrecht D, Gerth U. et al. Cross-talk between the general stress response and sporulation initiation in *Bacillus subtilis*—the σB promoter of spo0E represents an AND-gate. *Environ Microbiol* 2012;14:2741–56. 10.1111/j.1462-2920.2012.02755.x 22524514

[34] Reder A, Gerth U, Hecker M. Integration of σB activity into the decision-making process of sporulation initiation in *Bacillus subtilis*. *J Bacteriol* 2012;194:1065–74. 10.1128/JB.06490-11 22210769 PMC3294812

[35] Fujita M, Losick R. Evidence that entry into sporulation in *Bacillus subtilis* is governed by a gradual increase in the level and activity of the master regulator Spo0A. *Genes Dev* 2005;19:2236–44. 10.1101/gad.1335705 16166384 PMC1221893

[36] Molina-Santiago C, Pearson JR, Navarro Y. et al. The extracellular matrix protects *Bacillus subtilis* colonies from *Pseudomonas* invasion and modulates plant co-colonization. *Nat Commun* 2019;10:1919. 10.1038/s41467-019-09944-x 31015472 PMC6478825

[37] Grandchamp GM, Caro L, Shank EA. Pirated siderophores promote sporulation in *Bacillus subtilis*. *Appl Environ Microbiol* 2017;83:e03293–16. 10.1128/AEM.03293-16 28283524 PMC5411514

[38] Müller S, Strack SN, Hoefler BC. et al. Bacillaene and sporulation protect *Bacillus subtilis* from predation by *Myxococcus xanthus*. *Appl Environ Microbiol* 2014;80:5603–10. 10.1128/AEM.01621-14 25002419 PMC4178607

[39] Müller S, Strack SN, Ryan SE. et al. Predation by *Myxococcus xanthus* induces *Bacillus subtilis* to form spore-filled megastructures. *Appl Environ Microbiol* 2015;81:203–10. 10.1128/AEM.02448-14 25326308 PMC4272737

[40] Pérez-Lorente AI, Molina-Santiago C, de Vicente A. et al. Sporulation activated via σ W protects *Bacillus* from a Tse1 peptidoglycan hydrolase Type VI Secretion System effector. *Microbiol Spectr* 2023;11:e05045–22. 10.1128/spectrum.05045-22 36916921 PMC10100999

[41] Nair DVT, Venkitanarayanan K, Johny AK. Antibiotic-resistant *Salmonella* in the food supply and the potential role of antibiotic alternatives for control. *Food Secur* 2018;7:167. 10.3390/foods7100167 PMC6210005 30314348

[42] Stefanic P, Mandic-Mulec I. Social interactions and distribution of *Bacillus subtilis* pherotypes at microscale. *J Bacteriol* 2009;191:1756–64. 10.1128/JB.01290-08 19114482 PMC2648371

[43] Konkol MA, Blair KM, Kearns DB. Plasmid-encoded ComI inhibits competence in the ancestral 3610 strain of *Bacillus subtilis*. *J Bacteriol* 2013;195:4085–93. 10.1128/JB.00696-13 23836866 PMC3754741

[44] Stefanic P, Kraigher B, Lyons NA. et al. Kin discrimination between sympatric *Bacillus subtilis* isolates. *Proc Natl Acad Sci USA* 2015;112:14042–7. 10.1073/pnas.1512671112 26438858 PMC4653157

[45] Kraigher B, Butolen M, Stefanic P. et al. Kin discrimination drives territorial exclusion during *Bacillus subtilis* swarming and restrains exploitation of surfactin. *ISME J* 2022;16:833–41. 10.1038/s41396-021-01124-4 34650232 PMC8857193

[46] Chen Y, Chai Y, Guo J. et al. Evidence for cyclic Di-GMP-mediated signaling in *Bacillus subtilis*. *J Bacteriol* 2012;194:5080–90. 10.1128/JB.01092-12 22821967 PMC3430322

[47] Burton BM, Marquis KA, Sullivan NL. et al. The ATPase SpoIIIE transports DNA across fused septal membranes during sporulation in *Bacillus subtilis*. *Cell* 2007;131:1301–12. 10.1016/j.cell.2007.11.009 18160039 PMC2913279

[48] Spacapan M, Danevčič T, Stefanic P. et al. The ComX quorum sensing peptide of *Bacillus subtilis* affects biofilm formation negatively and sporulation positively. *Microorganisms* 2020;8:1131. 10.3390/microorganisms8081131 32727033 PMC7463575

[49] Koo BM, Kritikos G, Farelli JD. et al. Construction and analysis of two genome-scale deletion libraries for *Bacillus subtilis*. *Cell Syst* 2017;4:291–305.e7. 10.1016/j.cels.2016.12.013 28189581 PMC5400513

[50] Hoiseth SK, Stocker BAD. Aromatic-dependent *Salmonella* Typhimurium are non-virulent and effective as live vaccines. *Nature* 1981;291:238–9. 10.1038/291238a0 7015147

[51] Fields PI, Swanson RV, Haidaris CG. et al. Mutants of *Salmonella* Typhimurium that cannot survive within the macrophage are avirulent. *Proc Natl Acad Sci USA* 1986;83:5189–93. 10.1073/pnas.83.14.5189 3523484 PMC323916

[52] Valdivia RH, Falkow S. Bacterial genetics by flow cytometry: rapid isolation of *Salmonella* Typhimurium acid-inducible promoters by differential fluorescence induction. *Mol Microbiol* 1996;22:367–78. 10.1046/j.1365-2958.1996.00120.x 8930920

[53] Robijns SCA, Roberfroid S, Van Puyvelde S. et al. A GFP promoter fusion library for the study of *Salmonella* biofilm formation and the mode of action of biofilm inhibitors. *Biofouling* 2014;30:605–25. 10.1080/08927014.2014.907401 24735176

[54] Santiviago CA, Reynolds MM, Porwollik S. et al. Analysis of pools of targeted *Salmonella* deletion mutants identifies novel genes affecting fitness during competitive infection in mice. *PLoS Pathog* 2009;5:e1000477. 10.1371/journal.ppat.1000477 19578432 PMC2698986

[55] Wang S, Yang D, Wu X. et al. The ferric uptake regulator represses Type VI Secretion System function by binding directly to the *clpV* promoter in *Salmonella enterica* serovar Typhimurium. *Infect Immun* 2019;87:10–1128. 10.1128/IAI.00562-19 PMC6759306 31383745

[56] Dogsa I, Spacapan M, Dragoš A. et al. Peptide signaling without feedback in signal production operates as a true quorum sensing communication system in *Bacillus subtilis*. *Commun Biol* 2021;4:58. 10.1038/s42003-020-01553-5 33420264 PMC7794433

[57] Piggot PJ, Hilbert DW. Sporulation of *Bacillus subtilis*. *Curr Opin Microbiol* 2004;7:579–86. 10.1016/j.mib.2004.10.001 15556029

[58] Londoño-Vallejo JA, Fréhel C, Stragier P. *spoIIQ*, a forespore-expressed gene required for engulfment in *Bacillus subtilis*. *Mol Microbiol* 1997;24:29–39. 10.1046/j.1365-2958.1997.3181680.x 9140963

[59] Moore CM, Nakano MM, Wang T. et al. Response of *Bacillus subtilis* to nitric oxide and the nitrosating agent sodium nitroprusside. *J Bacteriol* 2004;186:4655–64. 10.1128/JB.186.14.4655-4664.2004 15231799 PMC438601

[60] Locke JCW, Young JW, Fontes M. et al. Stochastic pulse regulation in bacterial stress response. *Science* 2011;334:366–9. 10.1126/science.1208144 21979936 PMC4100694

[61] Levin PA, Losick R. Transcription factor Spo0A switches the localization of the cell division protein FtsZ from a medial to a bipolar pattern in *Bacillus subtilis*. *Genes Dev* 1996;10:478–88. 10.1101/gad.10.4.478 8600030

[62] Mutlu A, Trauth S, Ziesack M. et al. Phenotypic memory in *Bacillus subtilis* links dormancy entry and exit by a spore quantity-quality tradeoff. *Nat Commun* 2018;9:69. 10.1038/s41467-017-02477-1 29302032 PMC5754360

[63] Mutlu A, Kaspar C, Becker N. et al. A spore quality–quantity tradeoff favors diverse sporulation strategies in *Bacillus subtilis*. *ISME J* 2020;14:2703–14. 10.1038/s41396-020-0721-4 32724142 PMC7784978

[64] Sana TG, Flaugnatti N, Lugo KA. et al. *Salmonella* Typhimurium utilizes a T6SS-mediated antibacterial weapon to establish in the host gut. *Proc Natl Acad Sci USA* 2016;113:E5044–51. 10.1073/pnas.1608858113 27503894 PMC5003274

[65] Lories B, Roberfroid S, Dieltjens L. et al. Biofilm bacteria use stress responses to detect and respond to competitors. *Curr Biol* 2020;30:1231–1244.e4. 10.1016/j.cub.2020.01.065 32084407 PMC7322538

[66] Giannakopoulou N, Mendis N, Zhu L. et al. The virulence effect of CpxRA in *Citrobacter rodentium* is independent of the auxiliary proteins NlpE and CpxP. *Front Cell Infect Microbiol* 2018;8:320. 10.3389/fcimb.2018.00320 30280092 PMC6153362

[67] Yi Z, Wang D, Xin S. et al. The CpxR regulates Type VI Secretion System 2 expression and facilitates the interbacterial competition activity and virulence of avian pathogenic *Escherichia coli*. *Vet Res* 2019;50:1–12. 10.1186/s13567-019-0658-7 31126325 PMC6534853

[68] Cho T, Pick K, Raivio TL. Bacterial envelope stress responses: essential adaptors and attractive targets. *Biochim Biophys Acta*—*Mol Cell Res* 2023;1870:119387. 10.1016/j.bbamcr.2022.119387 36336206

[69] Hews CL, Cho T, Rowley G. et al. Maintaining integrity under stress: envelope stress response regulation of pathogenesis in gram-negative bacteria. *Front Cell Infect Microbiol* 2019;9:313. 10.3389/fcimb.2019.00313 31552196 PMC6737893

[70] Raivio TL, Silhavy TJ. Transduction of envelope stress in *Escherichia coli* by the Cpx two- component system. *J Bacteriol* 1997;179:7724–33. 10.1128/jb.179.24.7724-7733.1997 9401031 PMC179735

[71] Raivio TL, Popkin DL, Silhavy TJ. The Cpx envelope stress response is controlled by amplification and feedback inhibition. *J Bacteriol* 1999;181:5263–72. 10.1128/JB.181.17.5263-5272.1999 10464196 PMC94031

[72] Craig JE, Ford MJ, Blaydon DC. et al. A null mutation in the *Bacillus subtilis* aconitase gene causes a block in Spo0A-phosphate-dependent gene expression. *J Bacteriol* 1997;179:7351–9. 10.1128/jb.179.23.7351-7359.1997 9393699 PMC179685

[73] Schierstaedt J, Jechalke S, Nesme J. et al. *Salmonella* persistence in soil depends on reciprocal interactions with indigenous microorganisms. *Environ Microbiol* 2020;22:2639–52. 10.1111/1462-2920.14972 32128943

[74] Lazarevic V, Soldo B, Médico N. et al. *Bacillus subtilis* α-phosphoglucomutase is required for normal cell morphology and biofilm formation. *Appl Environ Microbiol* 2005;71:39–45. 10.1128/AEM.71.1.39-45.2005 15640167 PMC544238

[75] Klobutcher LA, Ragkousi K, Setlow P. The *Bacillus subtilis* spore coat provides ‘eat resistance’ during phagocytic predation by the protozoan *Tetrahymena thermophila*. *Proc Natl Acad Sci USA* 2006;103:165–70. 10.1073/pnas.0507121102 16371471 PMC1324984

[76] Zhao H, Msadek T, Zapf J. et al. DNA complexed structure of the key transcription factor initiating development in sporulating bacteria. *Structure* 2002;10:1041–50. 10.1016/S0969-2126(02)00803-1 12176382

[77] Molle V, Fujita M, Jensen ST. et al. The Spo0A regulon of *Bacillus subtilis*. *Mol Microbiol* 2003;50:1683–701. 10.1046/j.1365-2958.2003.03818.x 14651647

[78] Singh RP, Kumari K. Bacterial Type VI Secretion System (T6SS): an evolved molecular weapon with diverse functionality. *Biotechnol Lett* 2023;45:309–31. 10.1007/s10529-023-03354-2 36683130

[79] Pei TT, Kan Y, Wang ZH. et al. Delivery of an Rhs‐family nuclease effector reveals direct penetration of the gram‐positive cell envelope by a type VI secretion system inAcidovorax citrulli. *mLife* 2022;1:66–78. 10.1002/mlf2.12007 38818323 PMC10989746

[80] Higgins D, Dworkin J. Recent progress in *Bacillus subtilis* sporulation. *FEMS Microbiol Rev* 2012;36:131–48. 10.1111/j.1574-6976.2011.00310.x 22091839 PMC3237856

[81] De Vries YP, Hornstra LM, Atmadja RD. et al. Deletion of *sigB* in *Bacillus cereus* affects spore properties. *FEMS Microbiol Lett* 2005;252:169–73. 10.1016/j.femsle.2005.08.042 16171954

[82] Bartolini M, Cogliati S, Vileta D. et al. Stress-responsive alternative sigma factor SigB plays a positive role in the antifungal proficiency of *Bacillus subtilis*. *Appl Environ Microbiol* 2019;85:e00178–19. 10.1128/AEM.00178-19 30824454 PMC6495766

[83] Atrih A, Foster SJ. Analysis of the role of bacterial endospore cortex structure in resistance properties and demonstration of its conservation amongst species. *J Appl Microbiol* 2001;91:364–72. 10.1046/j.1365-2672.2001.01394.x 11473602

[84] Yang X, Liu H, Zhang Y. et al. Roles of Type VI Secretion System in transport of metal ions. *Front Microbiol* 2021;12:756136. 10.3389/fmicb.2021.756136 34803980 PMC8602904

