# Supplementary material for: Bacillus subtilis ensures high spore quality in competition with Salmonella Typhimurium via the SigB-dependent pathway
Source: ISME J. 2025 Mar 18;19(1):wraf052. doi: 10.1093/ismejo/wraf052 (PMC11994997; doi:10.1093/ismejo/wraf052)
Supplement: Supplementary_material_Podnar_et_al_2025_13_3_wraf052 [file supplementary_material_podnar_et_al_2025_13_3_wraf052.pdf]

SUPPLEMENTARY MATERIAL

Bacillus subtilis ensures high spore quality in competition with *Salmonella* Typhimurium via the SigB-dependent pathway

Eli Podnar[1], Kristina Dendinovic[1†], Tjaša Danevčič[1], Bram Lories[2], Eva Kovačec[1‡], Hans Steenackers[2], Ines Mandic-Mulec[1*]

[1]Department of Microbiology, Biotechnical Faculty, University of Ljubljana, Ljubljana, Slovenia
[2]Department of Microbial and Molecular Systems, Centre of Microbial and Plant Genetics (CMPG), KU Leuven, Leuven, Belgium

Running title: Spore quality as a competition strategy

*Corresponding author: Ines Mandic-Mulec, Department of Microbiology, Biotechnical Faculty, University of Ljubljana, Večna pot 111, Ljubljana 1000, Slovenia

ines.mandicmulec@bf.uni-lj.si

† present address: Department of Laboratory Medicine, Medical University of Vienna, Vienna, Austria

‡ present address: Agricultural Institute of Slovenia, Ljubljana, Slovenia

This file includes:

Tables S1 to S3

Figures S1 to S6

Supplementary Table 1: Strains used in this study

**Strain name**	**Background**	**Genotype**	**Reference**
***B. subtilis* strains**			
PS-216	/	WT	[1]
BM1097	PS-216	*amyE*::$P_{hyspank}$-*mKate2* (Cm)	[2]
BM1629	PS-216	*sacA*::P_{43}-*mKate2* (Kn)	[3]
BM1625	PS-216	*amyE*::P_{spoIIQ}-*yfp* (Sp)<br>*sacA*::P_{43}-*mKate2* (Cm)	[3]
BM1996	PS-216	*amyE*::P_{spo0A}-*yfp* (Sp)<br>*sacA*::P_{43}-*mKate2* (Kn)	This work
BKE24610	PS-216	*sigB*::*ery*	[4]
BM1930	PS-216	*sigB*::*ery*	This work
BM1931	PS-216	*sigB*::*ery*<br>*amyE*::$P_{hyspank}$-*mKate2* (Cm)	This work
BM1992	PS-216	*sigB*::*ery*<br>*sacA*::P_{43}-*mKate2* (Kn)	This work
BM2017	PS-216	*sigB*::*ery*<br>*amyE*::P_{spoIIQ}-*yfp* (Sp)<br>*sacA*::P_{43}-*mKate2* (Kn)	This work
BM1098	PS-218	*amyE*::$P_{hyspank}$-*mKate2* (Cm)	[2]
BM1094	PS-196	*amyE*::$P_{hyspank}$-*mKate2* (Cm)	[5]
BM1223	NCIB 3610	*amyE*::$P_{hyspank}$-*mKate2* (Cm)	[6]
***Salmonella enterica* serovar Typhimurium**			
ATCC 14028 GFP	ATCC 14028	*pFPV25 gfpmut3* (Amp)	[7]
SL1344	/	WT	[8]
SL1344 GFP	SL1344	*pFPV25 gfpmut3* (Amp)	[9]

SL1344 Δ*clpV*	SL1344	Δ*clpV* (Kn)	This work
SL1344 Δ*hcp*	SL1344	Δ*hcp* (Kn)	This work
SL1344 Δ*cpxA*	SL1344	Δ*cpxA* (Amp)	This work
SL1344 Δ*cpxP*	SL1344	Δ*cpxP* (Amp)	This work
SL1344 Δ*cpxR*	SL1344	Δ*cpxR* (Amp)	This work
SL1344 Δ*rpoE*	SL1344	Δ*rpoE* (Amp)	This work
SL1344 P_{clpV}-GFP*3*	SL1344	*pFPV25* P_{clpV}-*gfpmut3*	This work

Supplementary Table 2: Plasmids used in this study

**Plasmid name**	**Background**	**Genotype**	**Reference**
pEM1121	*E. coli* DH5α	*amyE*::P_{spo0A}-*yfp* (Sp, Amp)	This work
pKM3	*E. coli* DH5α	*amyE*::P_{spoIIQ}-*yfp* (Sp, Amp)	[10]
pMS17	*E. coli* DH5α	*sacA*::P_{43}-*mKate2* (Kn, Amp)	[3]

Supplementary Table 3: Oligonucleotides used in this study

**Oligonucleotide name**	**Sequence 5' → 3'**	**Reference**
P_{spo0A}-F (*Eco*RI)	GCGGAATTCCCGATCCAAGACTGTTGAAAG	This work
P_{spo0A}-R (*Hind*III)	CGCAAGCTTATGTAGTTAACAGGATTCACCCTTGCTAC	This work
5pL-sigB	GGCGTCACAGAATGCAGAAC	[4]
3pR-sigB	TTTGCTTCAGCGCCTTCTAATAC	[4]

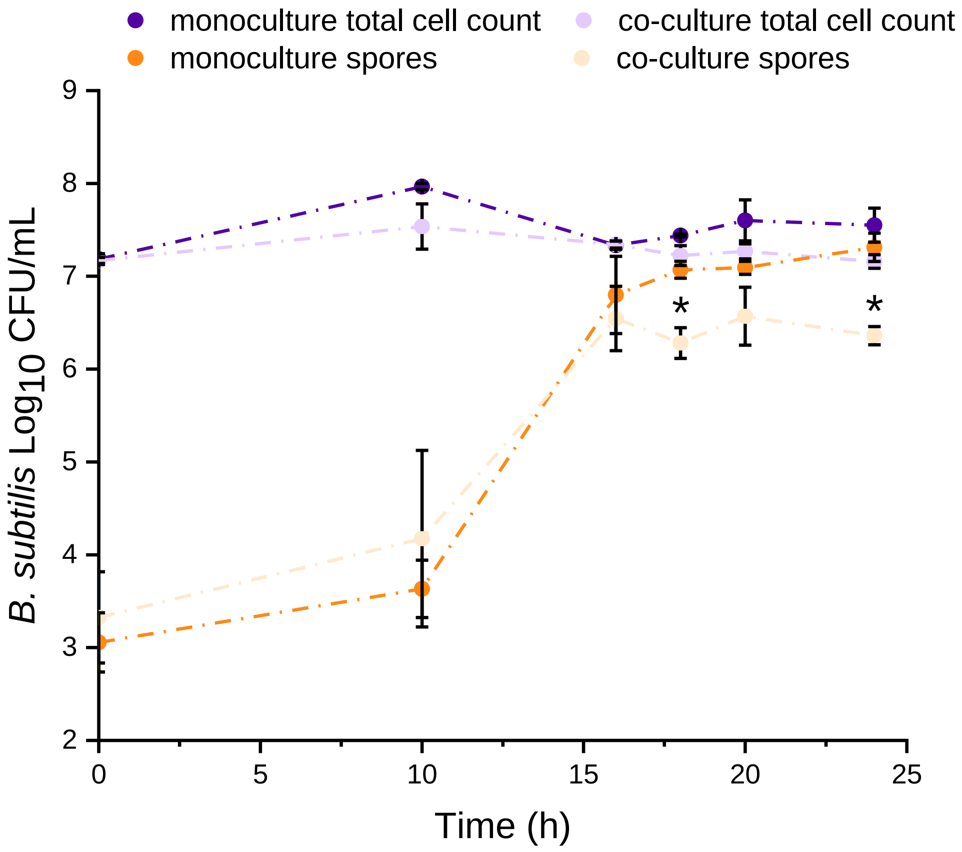

Supplementary Figure 1. ***B. subtilis*** **cell and spore counts at different time points.** Total cell counts and spore counts of *B. subtilis* PS-216 in monoculture and co-culture with *S.* Typhimurium SL1344 WT strain at the beginning of the experiment and after 10 h, 16 h, 18 h, 20 h and 24 h of incubation. Cell counts were determined at indicated time points and static incubation at 37°C in 1/20 TSB medium. Data are presented as mean values and error bars represent standard deviation of the mean values (n = 3 biologically independent experiments, each n performed in two technical replicates). Student's t-test was performed to compare the means between monoculture and co-culture, where statistically significant differences were determined (* $P < 0.05$).

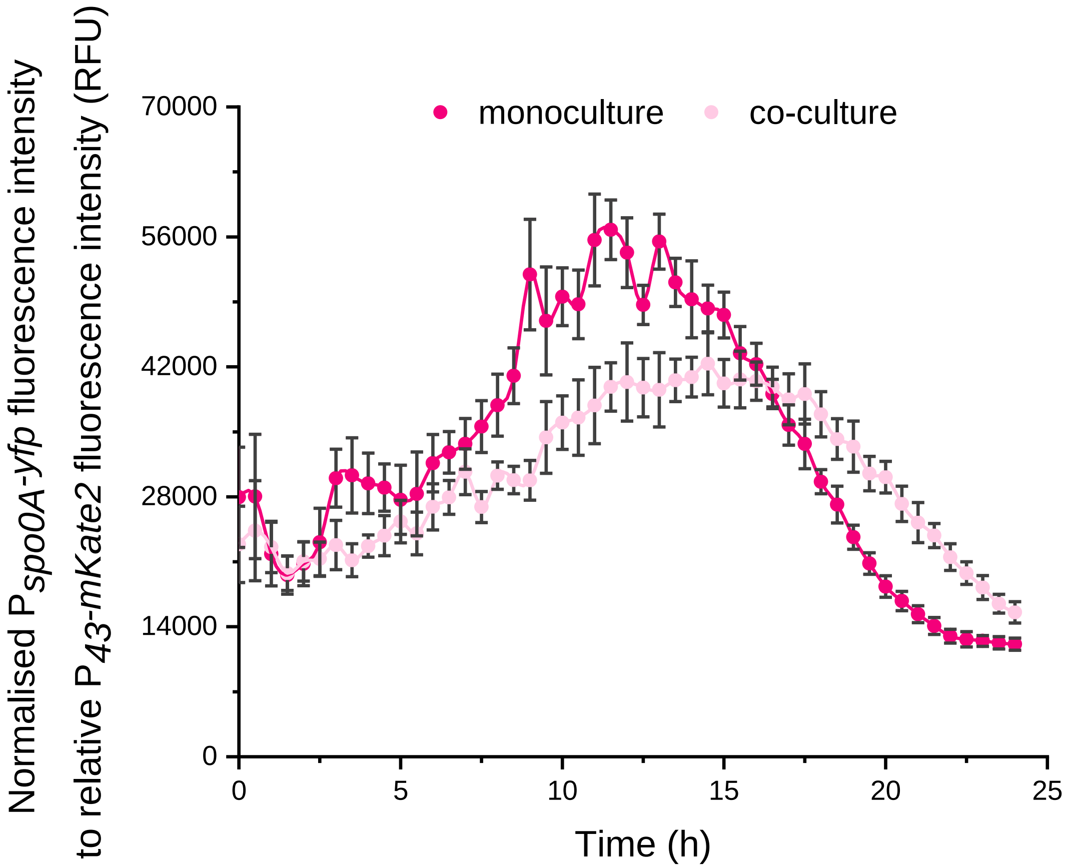

Supplementary Figure 2. The P$_{spo0A}$ activity of *B. subtilis* over 24 h. Bulk measurements of the P$_{spo0A}$-*yfp* promoter activity in *B. subtilis* PS-216 monoculture and co-culture with *S.* Typhimurium SL1344. Only one out of three biologically independent experiment is shown on the panel (performed in 6 technical replicates). Measurements were performed every half hour for 24 h in 1/20 TSB medium. Data are presented as mean values and error bars represent standard error of the mean values.

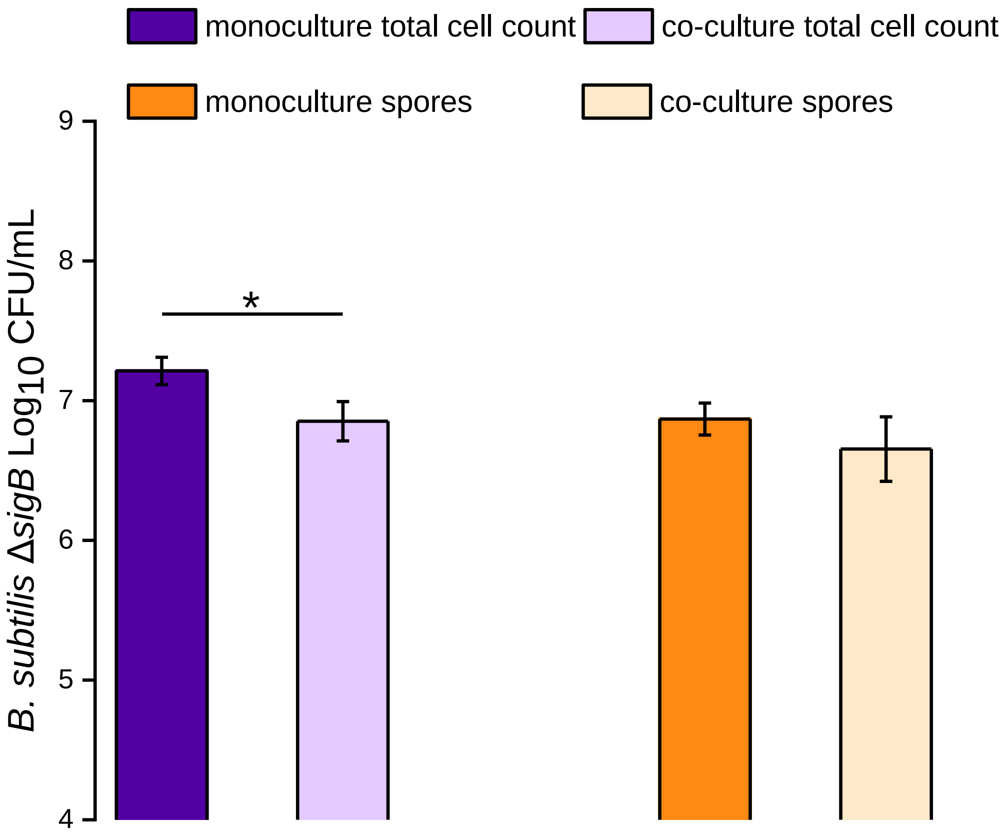

Supplementary Figure 3. ***B. subtilis*** **Δ*sigB*** **cell counts.** Total cell counts and spore counts of the *B. subtilis* Δ*sigB* mutant in monoculture and co-culture with *S.* Typhimurium SL1344 WT strain. Cell counts were determined after 24 h of static incubation at 37°C in 1/20 TSB medium. Data are presented as mean values and error bars represent standard deviation of the mean values (n = 3 biologically independent experiments, each n performed in two technical replicates). Student's t-test was performed to compare the means between monoculture and co-culture, where statistically significant differences were determined (* $P < 0.05$).

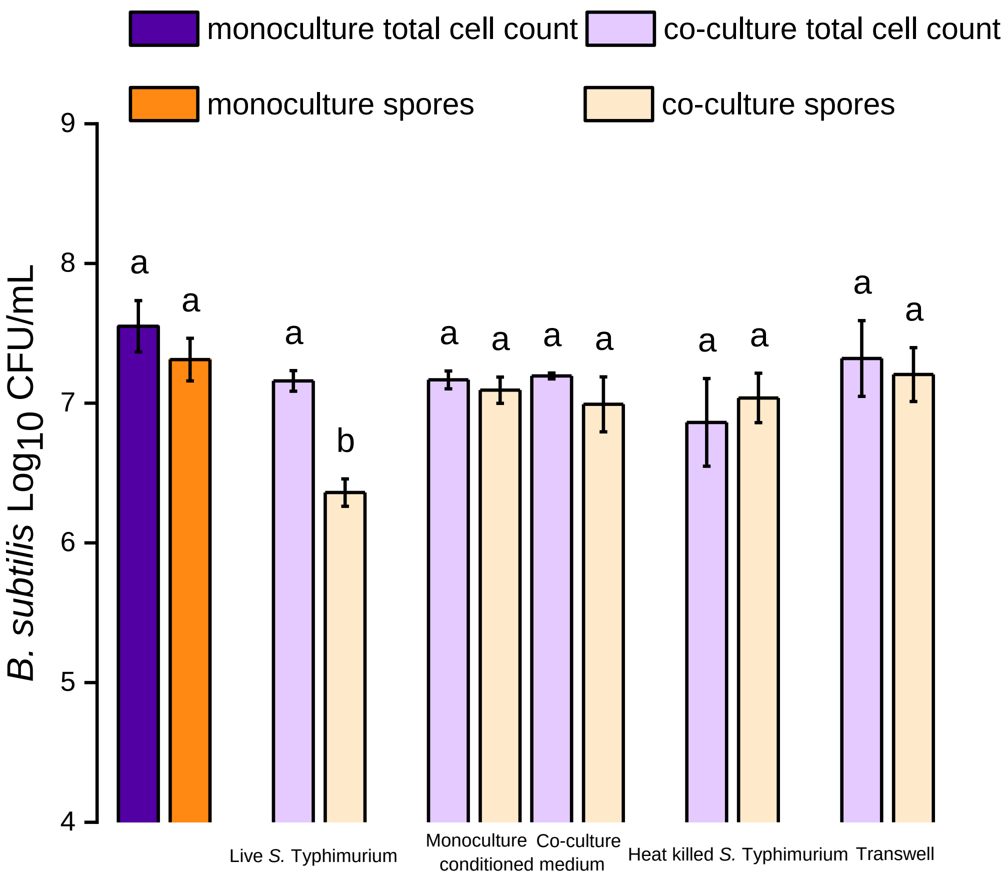

Supplementary Figure 4. ***B. subtilis*** **cell counts in co-cultures with conditioned medium, heat-killed** ***Salmonella*** **and in transwell system.** Total cell counts and spore counts of *B. subtilis* in monoculture and co-culture with *S.* Typhimurium SL1344 WT strain. Cell counts were determined after 24 h of static incubation at 37°C in 1/20 TSB medium. Data are presented as mean values and error bars represent standard deviation of the mean values (n = 3 biologically independent experiments, each n performed in two technical replicates). One-way ANOVA with Tukey's post hoc test ($P < 0.05$) was performed to compare the means of cell counts between different samples, where letters above the columns indicate a significant difference between samples.

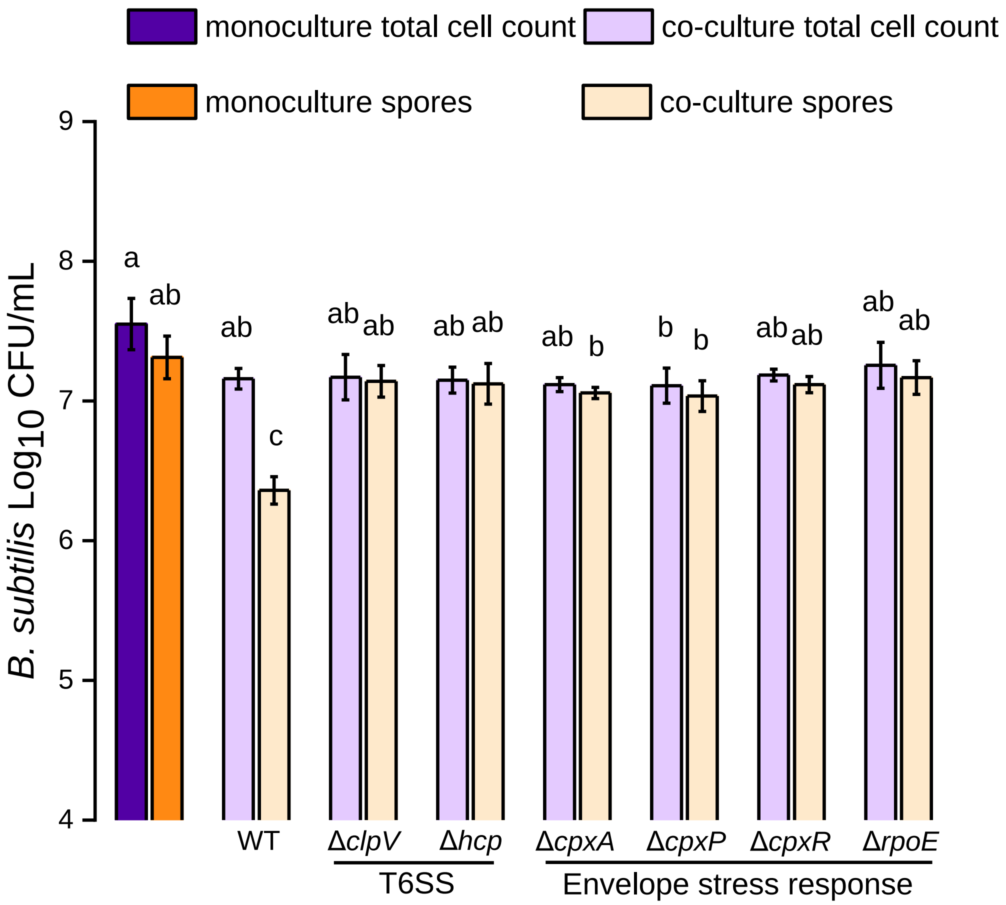

Supplementary Figure 5. *B. subtilis* cell counts in monoculture and co-cultures with *S.* Typhimurium mutants. Total cell counts and spore counts of *B. subtilis* in monoculture and co-culture with *S.* Typhimurium SL1344 WT strain. Cell counts were determined after 24 h of static incubation at 37°C in 1/20 TSB medium. Data are presented as mean values and error bars represent standard deviation of the mean values (n = 3 biologically independent experiments, each n performed in two technical replicates). One-way ANOVA with Tukey's post hoc test ($P < 0.05$) was performed to compare the means of cell counts between different samples, where letters above the columns indicate a significant difference between samples.

Methods for Supplementary Figure 6

Determination of the impact of iron on interspecies interactions

Mono- and co-cultures were prepared as described in the Materials and Methods section in the manuscript using 1/20 TSB medium supplemented with $FeCl_3$ in final concentrations of 0.05 mM and 0.1 mM. After 24 h of incubation total cell counts and spore fractions were determined as described in the the Materials and Methods section of the main manuscript.

Expression of P_{clpV} reporter strain and single cell measurements

To measure the promoter activity of the P_{clpV} at the single cell level static monocultures and co-cultures were grown in 1/20 TSB medium at 37°C. After 24 h, samples were disrupted by vigorous pipetting and vortexing. Subsequently, 100 000 *Salmonella* cells per sample were measured using a CytoFLEX S (Beckman Coulter). Gene expression was analysed using CytExpert software (Beckman Coulter). First, individual cells were identified based on forward and side scatter. Differentiation between *Salmonella* and *B. subtilis* cells was based on the red fluorescence of *B. subtilis* cells constitutively expressing the mKate2 protein. The activity of the P_{clpV} was quantified by measuring the fluorescence intensity of the green fluorescent protein.

Results and Discussion for Supplementary Figure 6

Because it is known that *B. subtilis* requires iron for sporulation [11], we hypothesized that competition between *S.* Typhimurium and *B. subtilis* will limit available iron and contribute to the observed sporulation impairment. To test this hypothesis, we supplemented 1/20 TSB medium with increasing concentrations of iron (i.e., 0.05 and 0.1 mM $FeCl_3$) and measured the sporulation frequency. In line with this prediction, we found that under iron supplementation, sporulation of *B. subtilis* was no longer impaired in co-culture with *S.* Typhimurium (Supplementary Figure 6A and 6B) suggesting that iron may play an indirect or direct role in this impairment.

Although Gram-positive and Gram-negative bacteria differ in their iron resistance due to differences in cell architecture, previous studies have shown that the iron concentrations used in our study are not toxic [12–14], which is consistent with the observation that neither 0.05 nor 0.1 mM $FeCl_3$ added to 1/20 TSB decreased cell counts of either species in monoculture (Supplementary Figure 6B and 6C). However, the addition of iron by passed sporulation impairment in co-culture, iron added to 1/20 TSB slightly decreased spore counts in monoculture, suggesting that it is not limited in 1/20 TSB.

Iron supplementation also decreased the cell counts of *S*. Typhimurium in co-culture suggesting a counterintuitive scenario, where the addition of iron increases competition for iron that negatively affects *S*. Typhimurium (Supplementary Figure 6C). The iron accessibility may be influenced by the medium composition. $FeCl_3$ interacts with phosphates, proteins, and amino acids in TSB medium [15]. Although in 1/20 TSB medium these interactions are likely to be reduced, $FeCl_3$ addition may still change iron bioavailability and consequently the interaction dynamics between the two species. For example, 1/20 TSB has a neutral pH, and at this pH, Fe^{3+} can precipitate as $Fe(OH)_3$ [16] making iron less accessible to both species. *B. subtilis* can overcome this limitation by using very efficient siderophores such as bacillibactin [14] and acquire iron more efficiently than *S.* Typhimurium, giving it a competitive

advantage over *S.* Typhimurium. Also, the pathogen's density is important for the sporulation impairment effect, hence, at low cell density *S.* Typhimurium fails to impair *B. subtilis* sporulation.

Alternatively, iron may inhibit T6SS expression [17], which could also restore sporulation. This idea has been experimentally tested using a P_{clpV} reporter strain of *S.* Typhimurium. However, results show that iron does not significantly alter the activity of the P_{clpV} promoter either in monoculture or in co-culture with *B. subtilis,* which disproves the prediction (Supplementary Figure 6D).

Results show that iron addition affects the dynamics of the interaction in co-culture, however, the underlying mechanisms remain unclear. Although intriguing, these findings are preliminary and require further investigation to establish a definitive interaction model involving iron supplementation.

Supplementary Figure 6

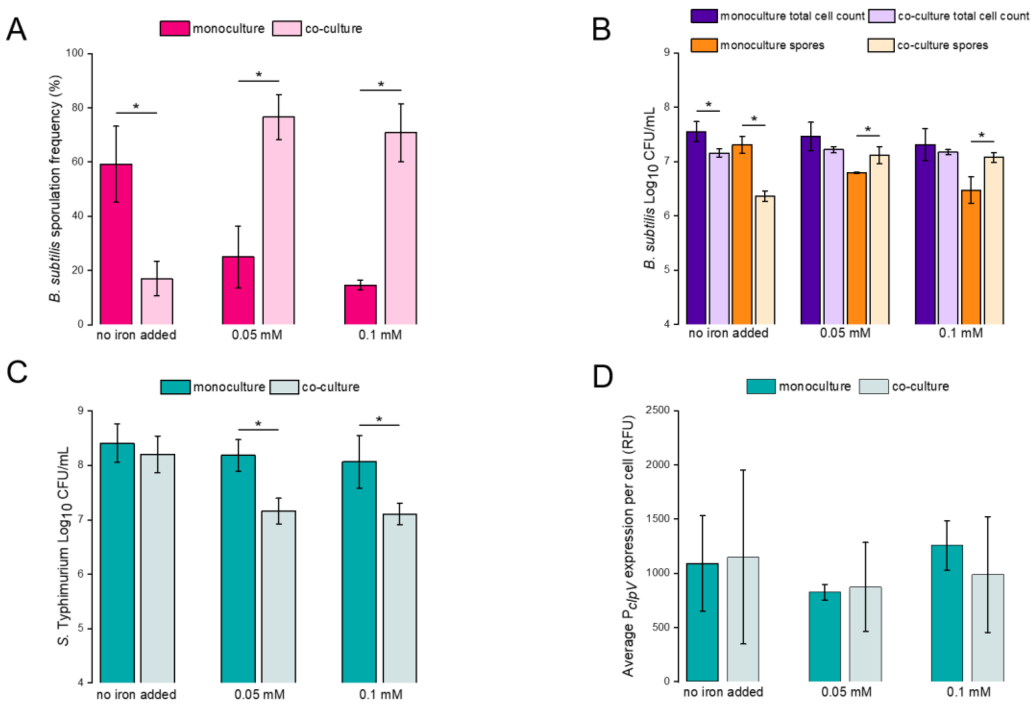

Supplementary Figure 6. Addition of iron restored the sporulation frequency of *B. subtilis* in co-culture with *S.* Typhimurium. (A) SF of *B. subtilis* PS-216 in co-culture with *S*. Typhimurium SL1344 strain at different iron concentrations. (B) *B. subtilis* cell counts in co-culture with *S.* Typhimurium at different iron concentrations. (C) *S.* Typhimurium cell counts in monocultures and co-cultures at different iron concentrations. Sporulation frequency and cell counts were determined after 24 h of static incubation at 37°C in 1/20 TSB medium without added iron and 1/20 TSB medium with different iron concentrations. (D) Single-cell expression of the P_{clpV} activity. P_{clpV} expression profiles in monocultures and cocultures of the *S.* Typhimurium SL1344 after 24 h of static incubation at 37°C in 1/20 TSB medium with different iron concentrations. Data are presented as mean values and error bars represent standard deviation of the mean values of three biologically independent experiments. Student's t-test was performed to compare the means of SF and cell counts between monoculture and co-culture (within the same medium), where statistically significant differences were determined (* $P < 0.05$).

References

1. Stefanic P, Mandic-Mulec I. Social interactions and distribution of *Bacillus subtilis* pherotypes at microscale. *J Bacteriol* 2009; **191**: 1756–1764.

2. Stefanic P, Kraigher B, Lyons NA, Kolter R, Mandic-Mulec I. Kin discrimination between sympatric *Bacillus subtilis* isolates. *Proc Natl Acad Sci U S A* 2015; **112**: 14042–14047.

3. Spacapan M, Danevčič T, Stefanic P, Porter M, Stanley-Wall NR, Mandic-Mulec I. The ComX quorum sensing peptide of *Bacillus subtilis* affects biofilm formation negatively and sporulation positively. *Microorganisms* 2020; **8**: 1131.

4. Koo BM, Kritikos G, Farelli JD, Todor H, Tong K, Kimsey H, et al. Construction and analysis of two genome-scale deletion libraries for *Bacillus subtilis*. *Cell Syst* 2017; **4**: 291-305.

5. Kraigher B, Butolen M, Stefanic P, Mandic Mulec I. Kin discrimination drives territorial exclusion during *Bacillus subtilis* swarming and restrains exploitation of surfactin. *ISME J* 2022; **16**: 833–841.

6. Chen Y, Chai Y, Guo J, Losick R. Evidence for cyclic Di-GMP-mediated signaling in *Bacillus subtilis*. *J Bacteriol* 2012; **194**: 5080–5090.

7. Robijns SCA, Roberfroid S, Van Puyvelde S, De Pauw B, Uceda Santamaría E, De Weerdt A, et al. A GFP promoter fusion library for the study of *Salmonella* biofilm formation and the mode of action of biofilm inhibitors. *Biofouling* 2014; **30**: 605–625.

8. Hoiseth SK, Stocker BAD. Aromatic-dependent *Salmonella* Typhimurium are non-virulent and effective as live vaccines. *Nature* 1981; **291**: 238–239.

9. Valdivia RH, Falkow S. Bacterial genetics by flow cytometry: Rapid isolation of *Salmonella* Typhimurium acid-inducible promoters by differential fluorescence induction. *Mol Microbiol* 1996; **22**: 367–378.

10. Burton BM, Marquis KA, Sullivan NL, Rapoport TA, Rudner DZ. The ATPase SpoIIIE transports DNA across fused septal membranes during sporulation in *Bacillus subtilis*. *Cell* 2007; **131**: 1301–1312.

11. Craig JE, Ford MJ, Blaydon DC, Sonenshein AL. A null mutation in the *Bacillus subtilis* aconitase gene causes a block in Spo0A-phosphate-dependent gene expression. *J Bacteriol* 1997; **179**: 7351–7359.

12. Xu Z, Mandic-Mulec I, Zhang H, Zhang N, Shen Q, Zhang R. Antibiotic bacillomycin d affects iron acquisition and biofilm formation in *Bacillus velezensis* through a Btr-mediated FeuABC-dependent pathway. *Cell Rep* 2019; **29**: 1192–1202.

13. Sorokina E V., Yudina TP, Bubnov IA, Danilov VS. Assessment of iron toxicity using a luminescent bacterial test with an *Escherichia coli* recombinant strain. *Microbiol* 2013, **82**: 439–444.

14. Lyng M, Jørgensen JPB, Schostag MD, Jarmusch SA, Aguilar DKC, Lozano-Andrade CN, et al. Competition for iron shapes metabolic antagonism between *Bacillus subtilis* and *Pseudomonas marginalis*. *ISME J* 2024; **18**: wrad001.

15. Liu X, Wu M, Li C, Yu P, Feng S, Li Y, et al. Interaction structure and affinity of zwitterionic amino acids with important metal cations (Cd^{2+}, Cu^{2+}, Fe^{3+}, Hg^{2+}, Mn^{2+}, Ni^{2+} and Zn^{2+}) in aqueous solution: a theoretical study. *Molecules* 2022, **27**: 2407.

16. Hem JD, Cropper WH. Survey of ferrous-ferric chemical equilibria and redox potentials. *USGPO* 1959; No. 1459-A.

17. Wang S, Yang D, Wu X, Yi Z, Wang Y, Xin S, et al. The ferric uptake regulator represses type VI secretion system function by binding directly to the *clpV* promoter in *Salmonella enterica* Serovar Typhimurium. *Infect Immun* 2019; **87**: 10–1128.
